# SpectrSeqTools: LC-MS/MS-Based De Novo Sequencing of Small Ribonucleic Acids with Post-Transcriptional Modifications

**DOI:** 10.1021/acs.analchem.6c02546

**Published:** 2026-09-25

**Authors:** Laura C. Kühle, Moshir Harsh, Shanice Jessica Hermon, Juan Magalang, Johannes Köster, Sidney Becker

**Affiliations:** † 28268 Max Planck Institute of Molecular Physiology, Otto-Hahn-Straße 11, 44227 Dortmund, Germany; ‡ Department of Chemistry and Chemical Biology, Technical University Dortmund, Otto-Hahn-Straße 6, 44227 Dortmund, Germany; § Bioinformatics and Computational Oncology, Institute for AI in Medicine (IKIM), Faculty of Medicine, 27170 University Hospital Essen, University of Duisburg-Essen, Girardetstraße 2, 45131 Essen, Germany; ∥ German Cancer Consortium (DKTK), University Hospital Essen, Hufelandstraße 55, Essen 45147, Germany

## Abstract

Post-transcriptional RNA modifications are key regulators of fundamental cellular processes. Yet, their precise biological functions remain largely unresolved. A major challenge is their reliable detection. While sequencing-based approaches are limited to a small subset of RNA modifications, mass spectrometry has the potential to directly identify various modified nucleotides within their sequence context. However, existing tools for nucleic acid analysis offer severely constrained functionality. Here, we introduce a set of algorithmic innovations implemented in SpectrSeqTools, an open-source LC-MS/MS-based software platform that enables fully automated de novo RNA sequencing together with comprehensive modification profiling of short RNA fragments directly from raw data. We further validated prediction accuracy and robustness using experimental and simulated data. As such, SpectrSeqTools effectively broadens the scope of nucleic acid mass spectrometry, enabling the read-out of up to 144 RNA modifications within their sequence context.

## Graphical abstract

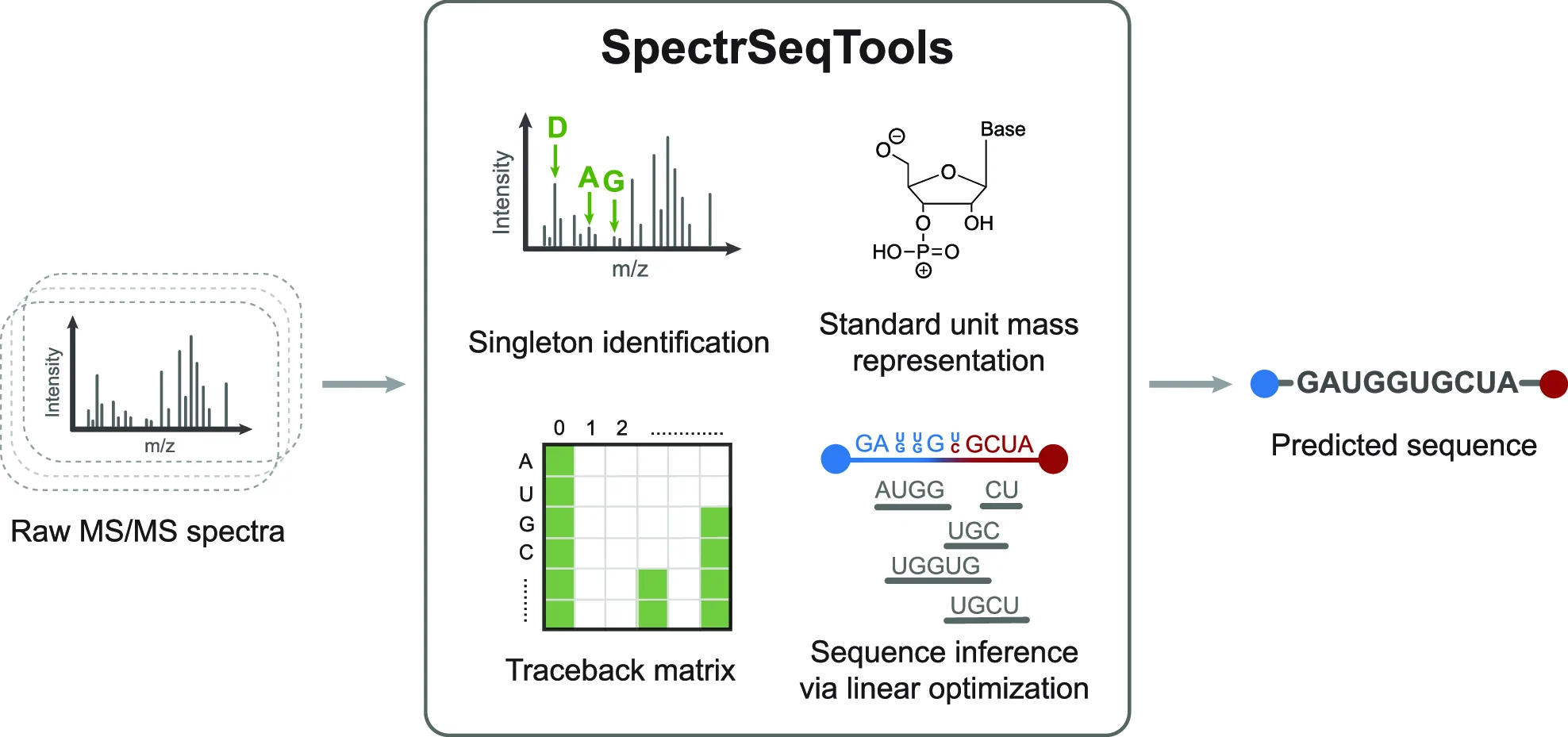

RNA molecules undergo a wide array of chemical modifications that influence their stability, structure, and function, thereby regulating gene expression.^1–3^ To date, over 170 distinct RNA modifications have been identified,^4^ with at least 50 present in the human transcriptome.^5^ Their precise spatiotemporal deposition is essential for normal human development, and its dysregulation is linked to various pathologies (Figure 1), highlighting their potential as diagnostic biomarkers.^3,6–10^ However, current sequencing technologies fail to capture the full spectrum of RNA modifications. Consequently, their distribution, dynamics, and interdependencies remain poorly understood.^11,12^ Addressing this gap is essential for elucidating their biological functions and uncovering disease mechanisms.

**Figure 1:**
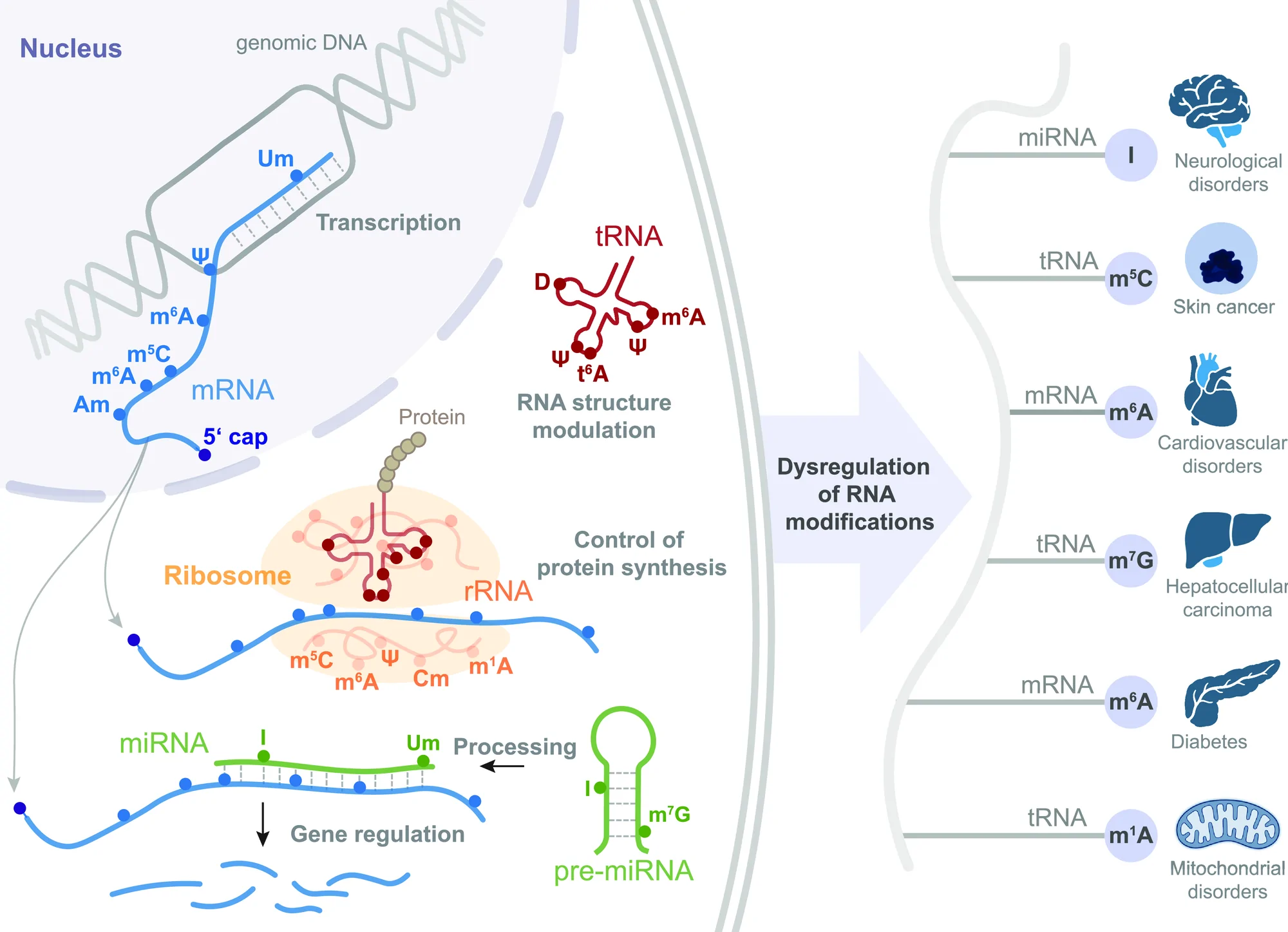
Biological functions and known examples for disease relevance of RNA modifications (so far challenging measurement of small RNAs implies that many occurring dysregulations are still unknown.^9,13,14^

While Nanopore-based sequencing allows for the direct detection of RNA modifications, it captures only a subset of the full modification landscape.^15–17^ Furthermore, because modification identification often relies on recognizing specific signal deviations against a reference, this approach is not entirely de novo and typically requires prior knowledge of the RNA sequence of interest.^18^ Other sequencing technologies generally lose modification information during RNA-to-cDNA conversion, though specialized protocols allow for the indirect detection of a small subset of the full modification space.^19–22^ While these platforms have significantly contributed to the characterization of epitranscriptomic signatures in mRNAs, rRNAs, and tRNAs^11,23,24^ they face severe limitations when profiling small regulatory RNAs^25–29^ such as miRNAs, piRNAs, siRNAs, and tRNA fragments (tRFs)−leaving their modification landscapes largely uncharacterized.

Liquid chromatography-tandem mass spectrometry (LC-MS/MS) is the gold standard for global RNA modification quantification. However, its potential to simultaneously and directly detect post-transcriptional modifications within their sequence context, including those inaccessible to sequencing-based methods^30,31^ has yet to be realized. In contrast to proteomics, nucleic acid LC-MS/MS analysis remains challenging: RNA’s limited chemical diversity (four nucleotides vs. 20 amino acids) hinders sequence identification, as illustrated by the 1 Da difference of cytidine and uridine. In contrast to the predictable, well-defined fragmentation of peptides, nucleic acids also generate complex fragmentation patterns with multiple ion series and extensive neutral losses.^32^ Frequent metal adduct formation further reduces sensitivity and complicates spectra.^33,34^ Collectively, these factors have hindered data interpretation and slowed the development of dedicated software for nucleic acid analysis relative to proteomics.

Most established analysis tools for MS/MS data, such as Ariadne^35^ and NASE,^36^ rely on database-dependent spectral matching. This creates an inherent reference bias toward known RNA sequences and characterized modification contexts. Such reliance is a severe limitation, not only due to incomplete or sparse reference information but also because static reference libraries fail to capture the dynamic fluctuation of RNA modifications across different cellular conditions or disease states. Commercial solutions like BioPharma Finder (Thermo Fisher Scientific Inc.) or BioPharma Compass (Bruker) similarly depend on predefined data input to confirm sequence information. This restricts their utility for characterizing RNA sequences or modification profiles beyond known references.

De novo RNA sequencing circumvents these limitations by directly interpreting fragmentation patterns without reference bias. Existing software for MS/MS-based de novo RNA sequencing falls into two categories: global fragmentation searches and local ladder-building methods. Early approaches focused on global searches and aimed at sequence assembly through simultaneous evaluation of all observed fragment ions.^37,38^ However, the need to compute a vast number of possible compositions to explain the intact mass limits scalability. Even RoboOligo,^39^ the most advanced tool, is restricted to sequences ≤12 nt and requires a small, predefined set of allowable modifications.

In contrast, ladder-building methods reconstruct sequences by identifying 5′- and 3′-terminal fragments and extending inward from both ends. This concept, first proposed by Ni et al.,^40^ was implemented in the SOS software^41^ for computer-assisted manual RNA sequencing with a limited number of modifications, but remains to be realized as a fully automated and scalable solution.

Independent of MS/MS-based approaches, Yuan et al.^42^ introduced MLC-seq, a de novo RNA sequencing method that employs end-labeling and acid hydrolysis to generate controlled degradation ladders for sequence reconstruction. Although innovative, reproducible acid hydrolysis and the risk of degrading labile RNA modifications under the harsh conditions may lead to incomplete or biased sequence profiles. Additionally, the lack of MS/MS fragmentation data makes resolving isobaric sequences difficult, a problem that is intensified in heterogeneous samples, where overlapping ladders obscure sequence reconstruction. Crucially, a software tool capable of the fully automated interpretation of such data sets remains unavailable.

Here, we introduce SpectrSeqTools, an LC-MS/MS-based software suite for de novo sequencing of short end-labeled RNAs. End-labeling allows the accurate classification of the MS/MS fragments into 5′- and 3′-terminal fragments and internal fragments providing structural information for sequence reconstruction. Both chemical^43^ and enzymatic^42^ RNA end-labeling are well-established techniques. Protocols and commercial kits are available. By combining end-labeling with several algorithmic innovations, SpectrSeqTools dramatically improves the scalability of MS/MS data interpretation, enabling fully automated determination of RNA sequences (so far up to 20 nt) while simultaneously capturing their post-transcriptional modification profile. Currently, the software incorporates a library of 144 RNA modifications. Yet, it operates independently of sequence databases, allowing for the unbiased determination of RNA sequences. We verified the robustness of our algorithm using both simulated and experimental data, demonstrating that SpectrSeqTools is highly accurate and robust to measurement noise.

## Methods

In this section, we describe the methodology behind SpectrSeqTools. For a less technical summary, we first provide an overview of the major algorithmic innovations (Section Algorithmic Innovations), followed by a formal description of the algorithmic components and their interplay (Section Algorithmic Details). Finally, we provide technical details on the measurement of the synthetic oligonucleotides for the evaluation described in the Results section (Section LC-MS/MS Data Acquisition).

### Algorithmic Innovations

With SpectrSeqTools, we introduce a compendium of novel algorithmic approaches for MS/MS-based RNA sequencing. Strikingly, they can be repurposed multiple times within SpectrSeqTools’ overall analysis workflow, enabling the efficient solving of diverse subproblems.

#### Standard Unit Mass Representation

The fragmentation step of MS/MS can lead to four different breakpoints (i.e. *fragmentation types*) between nucleotides (cf. Figure 2a), each of them leading to a different mass. Instead of considering all types at every algorithmic step, we normalize them away by considering nucleotides as standard units. The *standard unit* (*SU*) of a nucleotide represents it with c/y-fragmentation with both 5′- and 3′-ends in a charged state (cf. Figure 2c). While in reality the oxygen at the 5′-end would be protonated and a 2′,3′-cyclic phosphate would form at the 3′-end, the standard unit representation is advantageous because it allows arbitrary chaining of nucleotides without having to consider fragmentation-induced alterations to the overall fragment mass (cf. Figure 2d). We consider masses in their standard unit form (*SU-mass*) throughout the manuscript, if not explicitly stated otherwise. A detailed description of the approach and its implications can be found in Section Fragment Classification.

**Figure 2:**
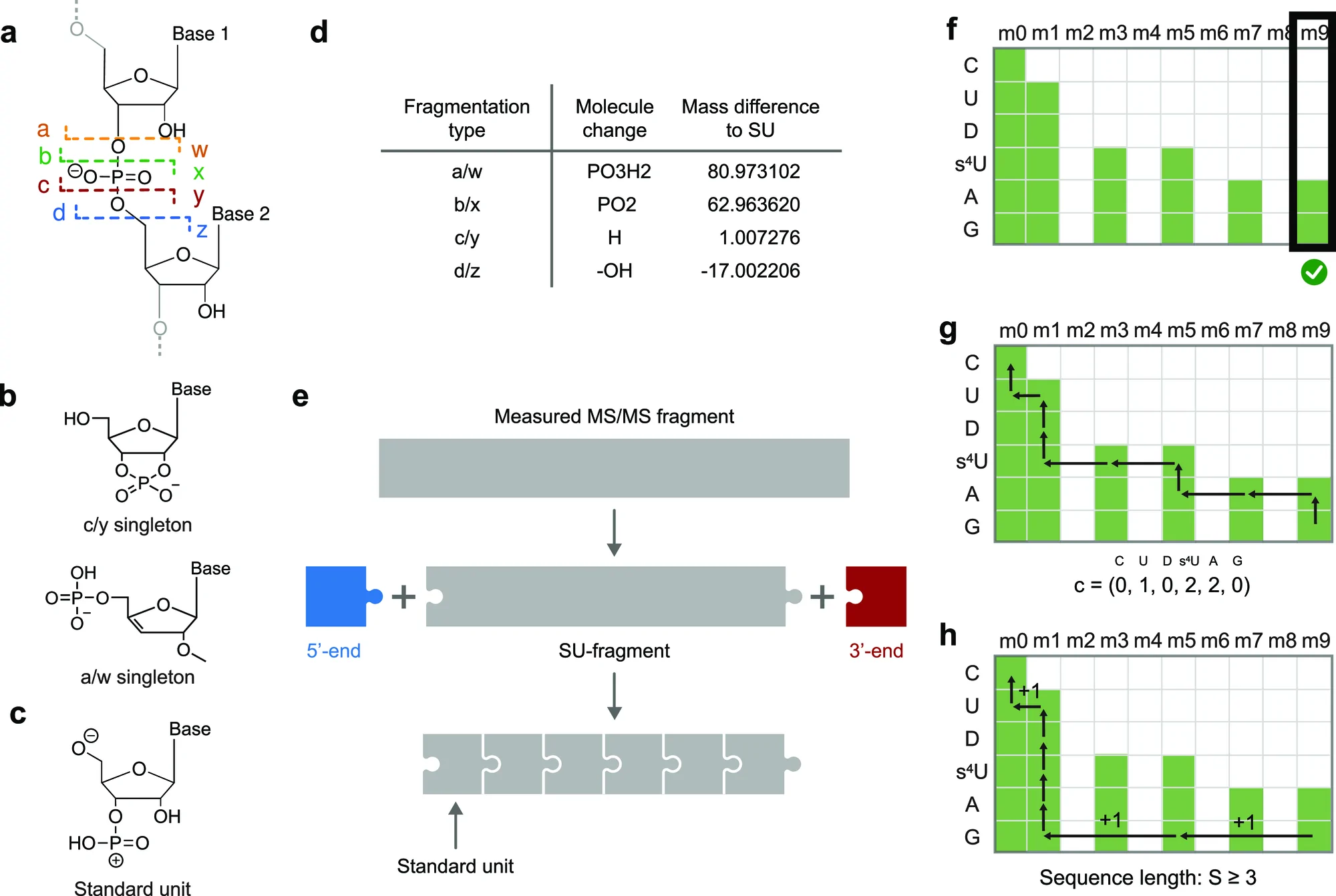
Highlights of algorithmic innovations in SpectrSeqTools. **a:** Visualization of MS/MS fragmentation types of RNA as defined by McLuckey et al.^44^ **b:** Representation of singletons generated from c/y- and a/w-fragmentation. **c:** Standard unit of any ribonucleotide. Note that−in contrast to the conventional definition of a nucleotide−both ends are charged, and the phosphate group is at the 3′-end. **d:** Mass differences for each fragmentation type in comparison to SU. Note that the values are given for the 5′-end and have to be negated for the 3′-end equivalent. **e:** Schematic depiction of the standardization process. Note that SU-fragments are standardized fragments as defined in Section Fragment Classification. **f:** Validity check with traceback matrix. **g:** Composition inference via traceback matrix. **h:** Minimum length estimation via traceback matrix.

#### Singleton Identification

SpectrSeqTools currently utilizes a library of 144 modified and unmodified nucleosides; however, only a subset of these typically appear in a sample sequence. To improve the computational efficiency, we reduce the search space by first automatically identifying which nucleotides are most likely to appear in the sequence. In addition, users can define custom modifications, enabling the identification of nonstandard modifications beyond those included in the default library. We coin the term *singleton* for an MS/MS fragment containing a single nucleotide. Singletons are typically single-base fragments generated through c/y-fragmentation; however, for modified nucleotides bearing a methyl group at the 2′-position of the ribose sugar, singleton masses are derived from a/w-fragmentation (cf. Figure 2b). The singletons of an MS/MS scan are identified by searching for MS/MS peaks that match the values in the reference nucleotide library. Reference nucleotides that do not have a feasible match with the MS/MS scans are excluded from the library, narrowing the set of candidate nucleotides used in the subsequent steps of the analysis. Section Preprocessing contains further details of this preprocessing method.

#### Traceback Matrix for Sequence Composition Assessment

Despite the final goal of inferring the sequence underlying the given mass observations, a central intermediate step is to determine the possible nucleotide compositions that could lead to a given mass. A composition can be seen as a multiset of nucleotides, the order of which is still unknown (and would also not change its mass). Compositions are required to determine lower and upper bounds for the length of the measured sequence (Figure 2h), to classify fragments into terminal and internal ones (cf. Section Fragment Classification), to sharpen the considered nucleotide alphabet (cf. Supplementary A), and to build a skeleton from ordered ladders of terminal fragments (cf. Section Skeleton Building). We present a *dynamic programming* algorithm that yields a universally applicable *traceback matrix* that allows the inference of any composition for a given mass in linear runtime (Figure 2g). Moreover, it allows us to determine the possibility of observing a given mass in constant runtime (Figure 2f). Importantly, the traceback matrix serves as an implicit and minimal representation of all possible compositions of all relevant masses for the currently investigated molecule. The traceback matrix is reused throughout the inference process of SpectrSeqTools.

#### Linear Optimization for Simultaneous Fragment Alignment and Sequence Inference

Each fragment yielded by the fragmentation process of MS/MS is a subsequence of the to-be-sequenced intact molecule. Their masses can serve as independent observations that together provide evidence for the RNA sequence of the intact molecule. We present a linear optimization approach that infers the sequence while simultaneously aligning all given fragments (both terminal and internal ones), yielding the most likely full sequence, as well as the position of each fragment within that sequence. Moreover, for each fragment, the approach yields the error between the theoretical mass of the fragment implied by its alignment and its observed mass. This novel consideration of internal fragments vastly increases the amount of available evidence and thus improves the accuracy and robustness of results. To constrain the search space, the optimization can be seeded with a skeleton sequence, which we infer via a ladder-building approach. The optimization is not only used to infer the final sequence of the measured molecule, but also to filter out fragments that exhibit too large errors when aligning them against the skeleton sequence, thereby efficiently cleaning up the data.

### Algorithmic Details

In this section, we describe the workings of SpectrSeqTools in detail, largely following the workflow depicted in Figure 3. The software is designed for LC-MS/MS data from end-labeled sequences and consists of four main steps, each contained in its own module: preprocessing (Section Preprocessing), fragment standardization and classification (Section Fragment Classification), skeleton computing via ladder building (Section Skeleton Building), and final sequence inference by linear optimization (Section Sequence Inference).

**Figure 3:**
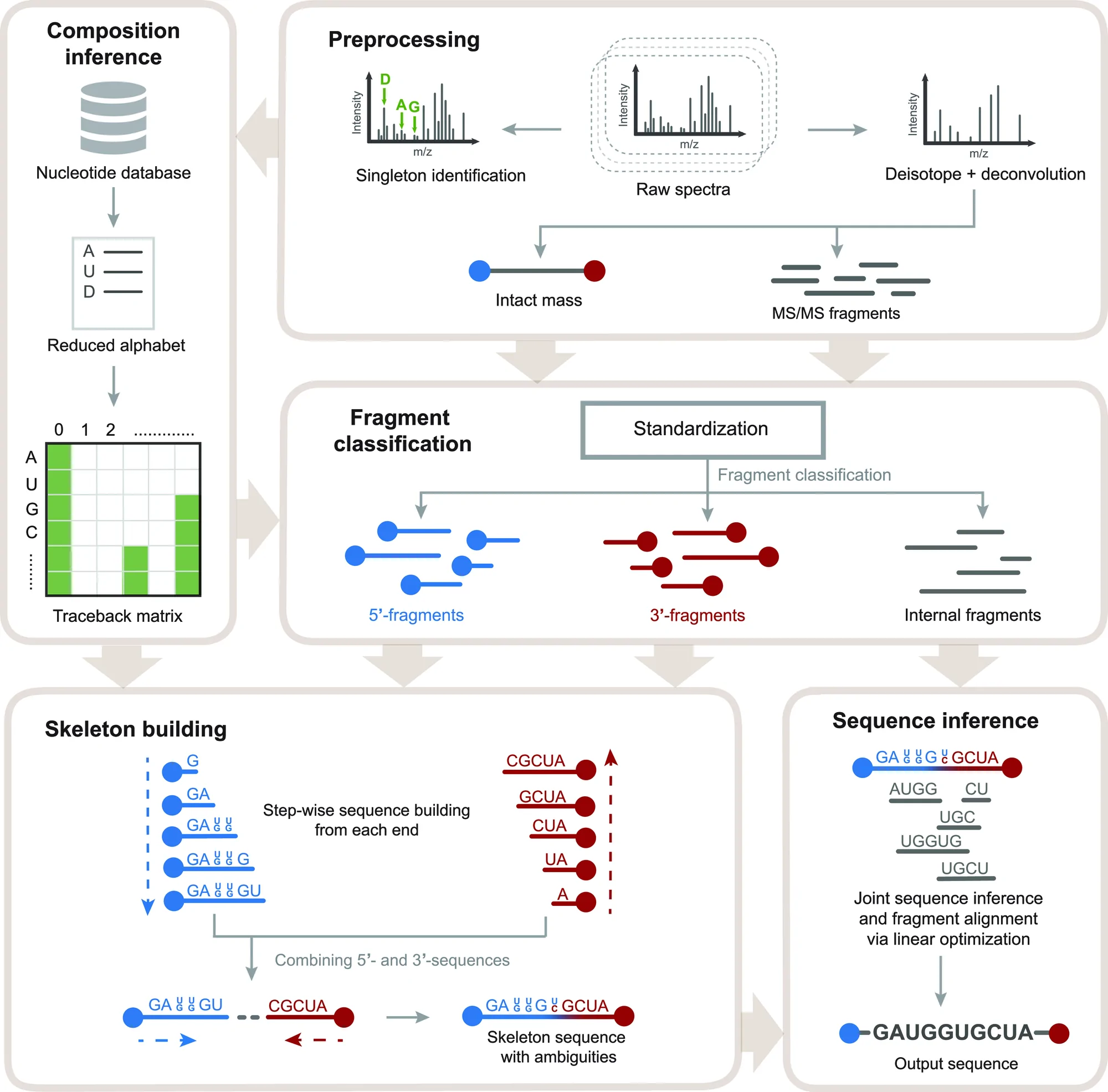
Schematic workflow depicting the individual modules in SpectrSeqTools. For simplicity, all filters are omitted. Furthermore, intact fragments are considered to be both 5′- and 3′-fragments, but otherwise not depicted.

In contrast to other MS-based RNA sequencing methods that first compute a full list of possible compositions and then filter out those with insufficient evidence in the measured MS/MS data, SpectrSeqTools only implicitly determines the compositions with a traceback matrix that is computed via dynamic programming (cf. Section Composition Inference) and has multiple applications, most prominently in the various filters used throughout the entire prediction process. For simplicity, all filter details are omitted in the workflow and instead described in Supplementary A.

#### Notation

We denote any length- or count-related variables with capital Latin letters (e.g., *N*), and capital calligraphy or Fraktur letters for sets, tuples, and matrices. The letters *k*, *i*, *j*, and *l* are used as index variables. The first three are consistently used for sequence positions, nucleotides, and fragments. For functions and constants, we utilize small Greek letters.

#### Preprocessing

In the following, we describe SpectrSeqTools’ preprocessing for Thermo Fisher LC-MS/MS data. Note that the same procedures apply for other manufacturers, and SpectrSeqTools is implemented in a way that allows straightforward extensions of its applicability.

The preprocessing of raw data consists of two independent parts: singleton identification and deconvolution. LC-MS/MS data are comprised of a precursor MS scan followed by its corresponding MS/MS scans. We define a singleton as an MS/MS fragment consisting of a single nucleotide. On the one hand, identifying singletons prior to the sequencing workflow allows us to reduce the possible sequence compositions that need to be considered. On the other hand, since MS/MS scans will be measured in *m*/*z* (mass-to-charge ratio) peaks, one must perform deconvolution to obtain neutral masses. The precursor MS scan is used to compute the intact sequence mass, while the MS/MS scans are used to obtain the singletons and fragment masses considered for the subsequent prediction steps. Details regarding deconvolution, deisotoping, and calculation of the intact sequence mass are found in Supplementary B.

For the singleton identification, we define $T=\left(t_{1},t_{2},...,t_{T}\right)$ , T∈N , to be a sequence of retention times, and $P_{t}=\left(p_{1},p_{2},...\right)$ to be the sequence of measured *m*/*z* values in an MS/MS scan at a retention time t∈T , in other words $P_{t}$ is a single MS/MS scan.

Singleton identification is performed by matching *m*/*z* peaks of an MS/MS scan with a reference *m*/*z* list of modified and unmodified nucleosides, obtained from both MODOMICS^4^ and the database underlying Ariadne.^35^ For each nucleoside mass, we add the mass of one phosphorus, two oxygens, and subtract one proton to obtain the *m*/*z* value of the singletons.

Let $Q=\left\{q_{1},q_{2},...,q_{Q}\right\}$ , Q∈N , be the set of reference *m*/*z* values of modified and unmodified nucleoside bases. A reference nucleoside q∈Q is considered to be a *singleton match* in a scan $P_{t}$ if it satisfies the following:

1 $$\exists p_{j}\in P_{t}:\frac{\left|p_{j}-q\right|}{p_{j}}<\tau_{\mathrm{singleton}}$$

The constant τ_singleton_ is a specified mass error tolerance (10 ppm by default, consistent with the expected mass accuracy of the used mass spectrometer). Figure 4a shows the matching results from a single MS/MS scan.

**Figure 4:**
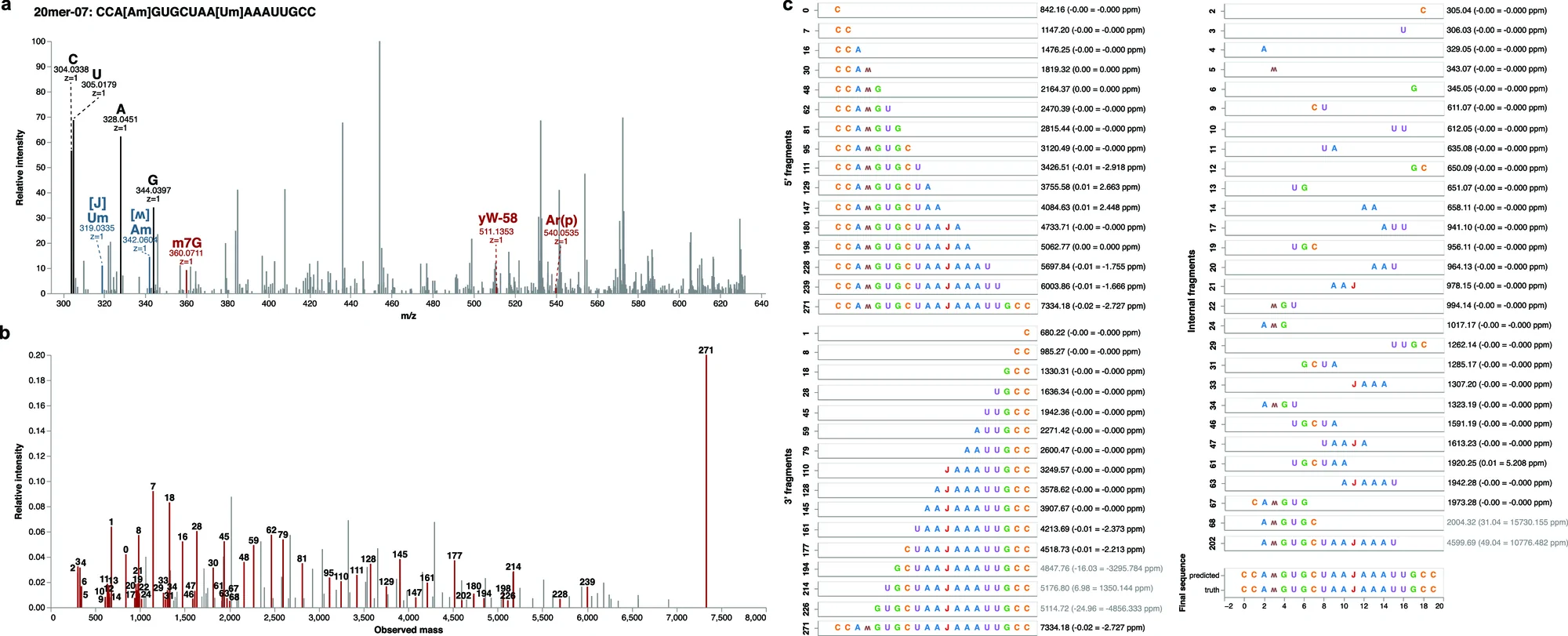
Output for the synthetic end-labeled RNA sequence 20mer-07. All figures were automatically generated with the Snakemake workflow and manually adapted. **a:** LC-MS/MS spectra with the identified singletons marked in black (canonical bases), blue (true positive modifications), and red (false positive modifications), respectively. **b:** Deconvoluted and deisotoped LC-MS/MS fragments with those used for the final sequence inference marked in red. **c:** Sequence assignment map, showing the predicted sequence for 5′-, 3′-, and internal fragments, their SU-masses, and the prediction error in ppm. Errors above the accepted tolerance τ_prediction_ (10 ppm by default) are marked in gray.

While false-negative singleton matches are−depending on the used collision energy−highly unlikely, false positives likely occur since raw measured *m*/*z* peaks may be noisy (cf. Supplementary C). However, using known RNA sequences, it has been observed that singletons that match across multiple MS/MS scans with retention times that are within a local range of each other are likely to be true positive matches. To reduce the number of false positives, we perform singleton matching outlined in Eq. 1 for all t∈T . For q∈Q , we define $T_{q}=\left\{t\in T\;|\;q\;\mathrm{is singleton match with}{\;P}_{t}\right\}$ .

We utilize density-based spatial clustering of applications with noise (DBSCAN)^45,46^ to group potential singleton peaks by their retention time: For $t\in T_{q}$ , we select a neighborhood of MS/MS scans in $T_{q}$ with radius ϵ_cluster_ centered at *t*. If the number of MS/MS scans within a ϵ_cluster_-neighborhood centered at *t* is greater than μ_cluster_, then *t* is called a *core point* ^46^ and all MS/MS scans within this neighborhood are indexed in the same cluster. If any of these neighbors are also core points, then their corresponding neighborhoods are also indexed in the same cluster. We use a version of DBSCAN optimized for 1D data sets (https://github.com/d-chambers/dbscan1d), and set to the default value of ϵ_cluster_ = 0.5 (this may also be estimated from the average time between MS/MS scans), and the minimum number of scans within a neighborhood to μ_cluster_ = 10.

To assess how well-clustered the retention times are for a given singleton match *q*, we use mean silhouette scores,^47^ which assign a value between −1 (retention times inappropriately clustered) and 1 (retention times appropriately clustered). A singleton match with a mean silhouette score <0 indicates sparse, uncertain clustering (i.e. sporadic signals over time), hence it is considered to be a false positive and subsequently rejected. Singleton matches with a silhouette score >0 are stored in a set to be used in the following steps.

#### Composition Inference

Based on the singletons determined during the preprocessing, we utilize dynamic programming to build a traceback matrix for efficient computing of compositions characterizing any given fragment mass.

Let $N=\left\{n_{1},n_{2},...,n_{N}\right\}$ , N∈N , be the set of unique masses that correspond to at least one nucleotide as determined during singleton identification (cf. Section Preprocessing); N is henceforth referred to as the *nucleotide alphabet*. For subsets of nucleotides with equal masses (cf. Supplementary D), we keep a single representative and denote alternative nucleotides with the same mass in the final reported sequence.

We build the traceback matrix D[i,j] , 0 ≤ *i* ≤ *N*, 0 ≤ *j* ≤ *D*, with rows corresponding to all unique nucleotide masses in the reduced alphabet and columns to possible total mass values. Because this implies all masses to be integer values, we first divide each mass by a precision ψ. In the following, all traceback-matrix-related masses (including *D*) are considered to be already divided by this precision. To set *D* (i.e. the highest total mass represented in D ) we multiply the mass of the heaviest nucleotide in the alphabet by the maximum sequence length to consider (which is 35 by default).

Let $M=\left\{m_{1},m_{2},...,m_{M}\right\}$ M∈N , be a set of fragment masses. For a given mass *m*, we define $C^{m}=\{\left(c_{1},c_{2},...,c_{N}\right)\;|\;c_{i}\in N_{0},m=\overset{N}{\underset{i=1}{\sum }}c_{i}n_{i}\}$ as the set of nucleotide compositions that (approximately according to the chosen precision ψ) result in mass *m*. Thereby, the *c*_*i*_ represent the number of copies of nucleotide *i* occurring in the respective composition. For each cell D[i,j] , we call it *reachable* if the given total mass *j* can be explained by any composition consisting only of nucleotides up to *i*, in other words, there is a $\left(c_{1},c_{2},...,c_{N}\right)\in C^{j}$ with $c_{i^{\prime }}=0$ for all *i*′ > *i*. Otherwise, it is *unreachable*.

To keep track of whether a reachable cell can be traced back to adding a nucleotide from another reachable cell in the same row or just carrying over the mass from another row, we define two bit flags that indicate possible leftward (indicated by the most significant bit) and upward movement (indicated by the least significant bit) in the traceback matrix, respectively. In the following, we represent such entries of *D* as base-2 integers (marked with a subscript _2_). For example, the entry $D\left[i,j\right]=11_{2}$ defines that both bits are set to 1, in other words a cell to the left $\left(D\left[i,j-\frac{n_{i}}{\psi }\right]\right)$ and the cell above (D[i−1,j]) are reachable. We further denote with ∧, ∨, and ¬ the bitwise AND-, OR-, and NOT-operators and with ≪ and ≫ the left and right SHIFT-operators.

To initialize the table, we set $D\left[0,0\right]=11_{2}=3$ and every other D[i,j]=0 . This corresponds to setting flags indicating that a total mass of 0 can be reached by selecting no nucleotides. We fill the table row-wise (starting at row *i* = 1) as depicted in Algorithm 1, considering one additional nucleotide in each outer iteration.
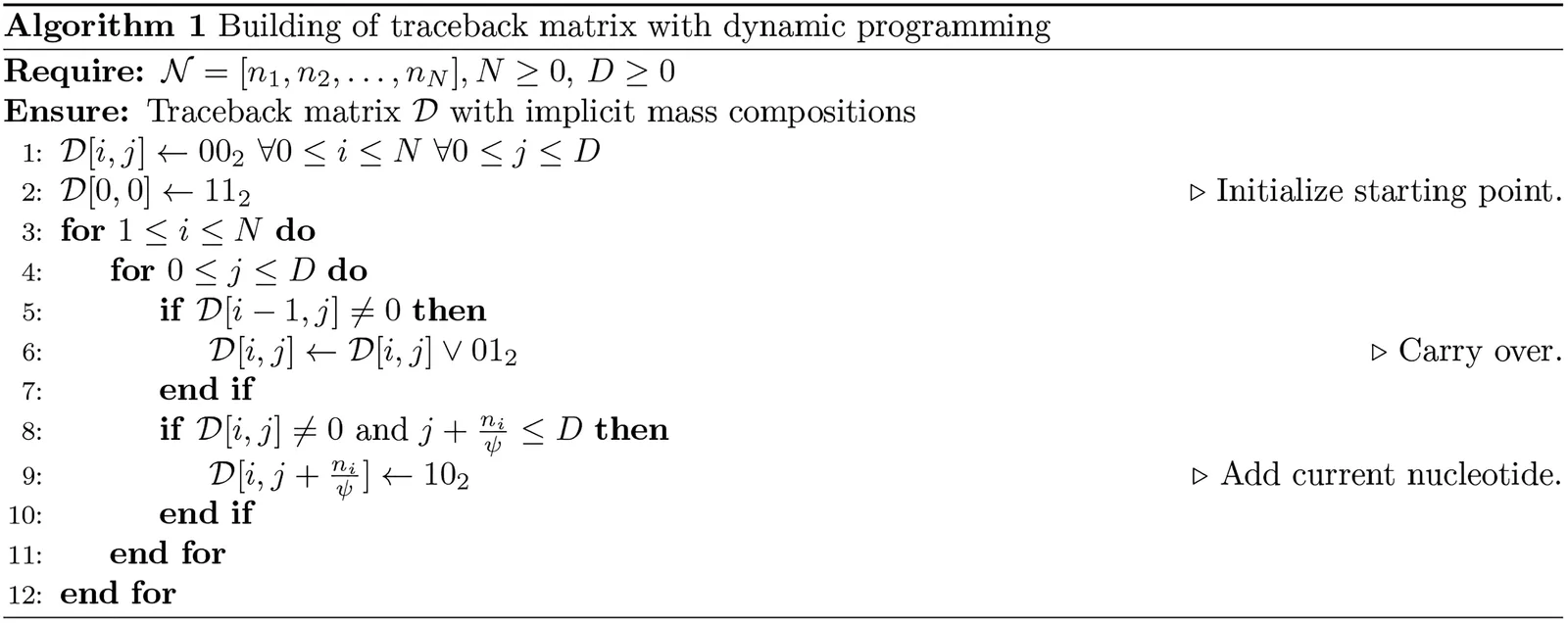


For each new row (i.e. nucleotide) *i*, we first carry over entries by setting the upward flag in each column with a reachable cell in the previous row. This represents the case that the corresponding mass can be explained without using nucleotide *i*. Then, we consider all reachable cells and−if possible−set the leftward flag for cells that can be reached by adding the mass of the nucleotide corresponding to the row.

With the full traceback matrix, we can then see whether a mass *m* has a valid composition by checking whether its cell in the corresponding column of the last row is reachable (D[N,m]≠0) . This look-up can be performed in constant time. Moreover, given a mass *m* ≤ *D*, any possible concrete composition can be obtained by performing traceback operations from cell D[N,m] to the origin of the traceback matrix (D[0,0]) in linear time, with O(N+σ(m)) in the worst case. Thereby,

σ(m)≔⌊m/(minN)⌋

is the maximum theoretically possible sequence length for a given mass *m*, consisting only of the lightest nucleotide.

Since each cell only requires space for two bits, we can save multiple cells in a single integer value. Let χ ∈ {4,8,16,32} be the desired compression rate (32 by default). Then, χ consecutive cells can be stored in the same 2χ-bit integer, occupying two bits each, starting from the two most significant bits. The traceback matrix D can then be emulated via a bit-compressed version $D^{\prime }$ by defining the following equivalences:

$$\begin{array}{ll} D\left[i,</j\right] & \;\equiv \;D\prime \;\left[i,</\left\lfloor j/w^{\prime }\right\rfloor \right]\gg 2\left(j\;\mathrm{mod}\chi \right)\wedge 11_{2}\\ D\left[i,</j\right]\leftarrow v & \;\equiv \;D\prime \;\left[i,</\left\lfloor j/w^{\prime }\right\rfloor \right]\leftarrow D\prime \;\left[i,</\left\lfloor j/w^{\prime }\right\rfloor \right]\\ & \;\wedge \neg \left(11_{2}\ll 2\left(j\;\mathrm{mod}\chi \right)\right)\vee \left(v\ll 2\left(j\;\mathrm{mod}\chi \right)\right) \end{array}$$

with *v* being a two-bit value that shall be set in the respective entry of D . In other words, for reading a cell value, the correct column in $D^{\prime }$ can be found by division with the compression rate χ, and the correct pair of bits can be reached by shifting to the right, followed by masking all but the two least significant bits. Writing a cell value works similarly, but here one has to first delete the corresponding bits by performing (i) a bit-wise AND-operation with a bitvector that has only the two bits where *v* shall be put as 0 and (ii) a bit-wise OR with the given value *v* shifted to the correct position. The reason for (i) is to clear the bits at the correct position in the bitvector in $D^{\prime }$ such that we can then update them with *v* in (ii). This reduces the memory complexity of the traceback matrix to

O(N·⌈D/χ⌉).

#### Fragment Classification

To simplify the table look-ups further, we introduce a standardization technique crucial to the workings of SpectrSeqTools, which we call *s*tandard units. The standard unit of a (modified or unmodified) base is defined as its nucleotide with c/y-fragmentation^44^ in a charged state as introduced in Section Algorithmic Innovations.

We used end-labeled oligonucleotides, with *m*_5′‑tag_ and *m*_3′‑tag_ being the masses of the label attached to the respective sequence end (relative to a SU-mass). These tags help to distinguish terminal from internal fragments. Further, let $F_{5^{\prime }}\in R^{4}$ and $F_{3^{\prime }}\in R^{4}$ be tuples of mass differences between an SU-fragment and ions for all possible fragmentation types, respectively. In theory, the mass difference between an observed fragment and the corresponding sequence in standard units either stems from noise or corresponds to a combination of the above (i.e. *fragmentation-related masses*). This conceptualization of fragments as a combination of standard units and fragmentation-related masses allows an early classification based on fragmentation type. Because all possible fragmentation-related masses are known (cf. Figure 2e), we can simply remove them and eliminate the need to consider them in each step of the actual prediction.

For simplicity, we coin the term standard unit *fragments (SU-fragments)* for fragments, from which the fragmentation-related mass (including end labels) has already been removed.

Their mass can thus be fully explained by the standard units of the nucleotides in the underlying sequence.

In the next stage of SpectrSeqTools, we hence aim to remove the mass-related uncertainty stemming from the unknown fragment class as depicted in Figure 2d. We thereby differentiate between *intact fragments* (contain both end labels and thus span the whole sequence), *5′-* and *3′-fragments* (contain only their respective label, but not the other), and *internal fragments* (contain no labels), which all have distinct, class-specific mass differences from an SU-fragment. For consistency, we refer to intact fragments as such, even though technically they are not fragments but the intact, nonfragmented sequence.

Generally, it is not trivial to determine the class of a given fragment, but false classification would affect the accuracy of the sequence prediction significantly. To avoid making assumptions, we favor a conservative approach that first considers every possible class for each fragment as follows:

2 $$M_{\mathrm{intact}}=\left\{m-m_{5^{’}‐\mathrm{tag}}-m_{3^{’}‐\mathrm{tag}}\;|\;m\in M\right\}$$

3 $$M_{5^{’}}=\left\{m-m_{5^{’}‐\mathrm{tag}}-f\;|\;m\in M,f\in F_{3^{’}}\right\}$$

4 $$M_{3^{’}}=\left\{m-f-m_{3^{’}‐\mathrm{tag}}\;|\;m\in M,f\in F_{5^{’}}\right\}$$

$$M_{\mathrm{internal}}=\left\{m-f_{1}-f_{2}\;|\;m\in M,f_{1}\in F_{3^{’}},f_{2}\in F_{5^{’}}\right\}.$$ 5

Each class contains SU-fragments gained by subtracting class-specific masses, respectively. For this work, we solely focus on the most abundant fragmentation type and thus exclusively consider c/y-fragmentation, which corresponds to generating at most one SU-fragment for each class-fragment combination. Note that we keep track of the origin of each SU-fragment to reduce the number of superfluous copies per original fragment as we go along. As a first step, the subsequent application of multiple filters removes all copies whose combination of standard unit mass and classification does not match the intact mass or cannot be reasonably explained by any composition (cf. Supplementary A).

#### Skeleton Building

The third stage is the construction of a so-called *skeleton*, a basic sequence that may still contain positions with ambiguities. For this stage, intact fragments are considered as both 5′- and 3′-fragments. First, we only consider 5′-fragments, assuming that all selected fragments correspond to a prefix of the sequence. Ordering them by mass in ascending order, we can reconstruct the sequence by utilizing the traceback matrix to find all nucleotide compositions (cf. Section Composition Inference) that explain the mass difference between two successive masses within a certain threshold. For speed-up and higher accuracy, fragments with similar mass are binned together (tolerance τ is set to 10 ppm by default; each fragment is represented by its mass). The first bin, $B_{0}$ , contains−by convention−a single theoretical empty fragment with mass 0.0.

After binning, the *5′-skeleton sequence*

$$S=\left(s_{1},s_{2},...,s_{S}\;|\;s_{k}\subseteq N\right)$$

can be determined iteratively. As the sequence length is at this point still unknown, we estimate it as an upper bound $S≔\sigma \left(m_{\mathrm{intact}}\right)$ , inferred from the intact mass (cf. Section Composition Inference). Each *s*_*k*_ represents the set of nucleotides (represented by their masses) that are considered possible at the respective position. Thereby, we track the next positions in S to determine the ascendingly ordered position set *P*. Initially, we set each $s_{k}≔N$ and *P* := {1}, representing that each position is still undetermined and the next position to consider is the first one. Ladder building is then performed by iteration through the ordered bins: For two successive bins $B_{1},B_{2}$ , SpectrSeqTools applies backtracking in the traceback matrix to compute

$$C=\underset{m_{1}\in B_{1},m_{2}\in B_{2}}{\cup }C^{m_{2}-m_{1}},$$

in other words, the set of all compositions for any mass difference between a fragment pair from different bins. If this set is empty, $B_{2}$ is removed, and all fragments contained therein are rejected (i.e. considered as wrongly classified as 5′-fragments). Otherwise, at least one composition was found and can be used to update the skeleton sequence. For the update, we first order the compositions *C* by ascending length and then consider each value *p* ∈ *P* in the position set. For each composition *c* = (*c*_1_,···,*c*_*N*_) ∈ *C*, let $\left|c\right|≔\overset{N}{\underset{i=1}{\sum }}c_{i}$ be its length and let ν­(*c*) := {*n*_*i*_ | *c*_*i*_ > 0, *i* = 1,···, *N*} be its set of contained nucleotides. From each position *p* ∈ *P* and each composition *c* ∈ *C*, we update |*c*| subsequent positions in the skeleton sequence (i.e. *s*_*p*+*l*_, *l* = 0,···, |*c*|-1). For each update, we distinguish between two cases: If (i) the composition is a subset or equal to the nucleotide set at position *p* + *l* in the skeleton (ν­(*c*) ⊆ *s*_*p*+*l*_), we restrict the position to *s*_*p*+*l*_ := ν­(*c*). Otherwise (ii) we set the position to the union of the nucleotides in the composition or previously recorded, that is *s*_*p*+*l*_ := ν­(*c*) ∪ *s*_*p*+*l*_. The rationale is the following: In case (i), the composition sharpens a previous estimate; in case (ii), the composition provides an alternative explanation, thus making the respective position more ambiguous. Since the order of nucleotides in the composition is unknown, it updates each position from *p* on, guided by its total length |*c*| (note that a composition may contain multiples of the same nucleotide). Lastly, the set of positions is updated to contain the viable direct successors after all considered positions, in other words P≔{p+|c||c∈C,p∈P,p+|c|≤S} . If this new position set is empty, all fragments in the remaining bins are rejected.

We proceed analogously for 3′-fragments and report the result in reverse order to gain the *3′-skeleton sequence*. For a correct combination of the two reconstructed ladders into a joint skeleton sequence, the sequence length S has to be determined. Note that up to this point, it was sufficient to use a rough upper bound based on dividing the intact mass *m*_intact_ by the lightest nucleotide mass as a sequence length estimate, but for the combined skeleton, a singular value has to be selected.

To restrict the considered length values, we leverage the intact mass in two ways: First, we define the range of length candidates by the composition lengths $\left\{|c|\;|\;c\in C^{m_{intact}}\right\}$ for the intact mass obtained via the traceback matrix. For each length candidate, we combine the two skeleton sequences by iterating through all positions and choosing the intersection if it is not empty and the union otherwise. Then, for a given combined skeleton sequence S , we determine $R_{S}=\left[\underset{s\in S}{\sum }\min\left(s\right),\underset{s\in S}{\sum }\max\left(s\right)\right]$ as the range between the minimum and maximum mass the sequence could exhibit by selecting at each position the lightest and heaviest nucleotide, respectively. We reject all length candidates, for which the intact mass does not fall into this range within an error margin (i.e. if $\left[m_{\mathrm{intact}}-\epsilon_{\mathrm{intact}},m_{\mathrm{intact}}+\epsilon_{\mathrm{intact}}\right]\cap R_{S}=Ø$ ).
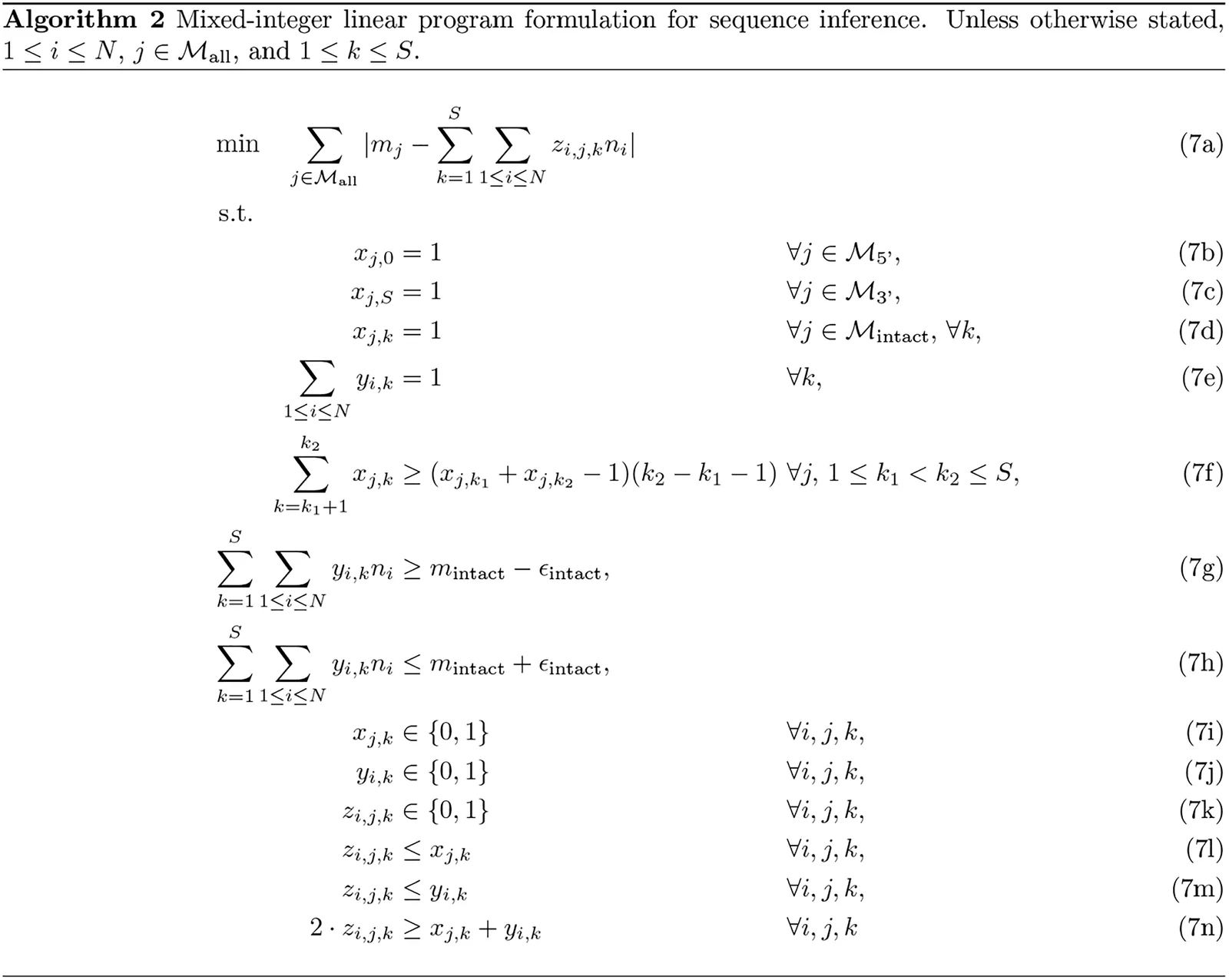


If all length candidates are rejected, we deem the whole prediction insufficient and report an empty sequence. Otherwise, the skeleton with the highest score for its corresponding length is selected, and we set S to be of its length. The considered score is the normalized sum of a Jaccard-based score at each position. For a given position, let $N_{3\prime }$ , $N_{5\prime }$ be the set of considered nucleotides for the 3′- and 5′-skeleton, respectively. We define the score at this position as

6 $${\mathrm{score}}_{\mathrm{Jaccard}}=\left\{ \begin{array}{ll} 1, & \mathrm{if}\;N_{3^{’}}\subseteq N_{5^{’}}\;\mathrm{or}\;N_{5^{’}}\subseteq N_{3^{’}}\\ \frac{\left|N_{3^{’}}\cap N_{5^{’}}\right|}{\left|N_{3^{’}}\cup N_{5^{’}}\right|}, & \mathrm{otherwise}, \end{array}\right.$$

representing a perfect score if the sets will be merged in the combined skeleton (if one is a subset of the other), and the Jaccard index (i.e. their agreement) otherwise.

#### Sequence Inference

The fourth stage aims to resolve remaining ambiguities in the skeleton. Ambiguities in the skeleton can occur when the mass difference between two nucleotides is so small that it falls within the tolerance τ_prediction_, when a composition contains more than one nucleotide that cannot be conclusively determined from either end, or when no agreement can be found between the two ladders at a specific position.

We formulate the resolution as a linear optimization problem to simultaneously align the up to now unused internal fragments and select the best-fitting nucleotide in each ambiguous position in the skeleton. In linear optimization, we define the problem in the form of an objective function (i.e. the term to optimize) and linear constraints. Here, the objective function aims to minimize the sum of differences between the measured mass of a fragment and the theoretical mass it would have based on its alignment in the skeleton. An example constraint is that all positions in a fragment have to be continuously aligned. The optimized result is the final sequence.

Let $M_{\mathrm{all}}=M_{\mathrm{intact}}\cup M_{5^{’}}\cup M_{3^{’}}\cup M_{\mathrm{internal}}$ be the set of all remaining fragments. Due to the filtering steps (cf. Supplementary A), each raw fragment should now have been assigned an unambiguous class (or have been removed entirely) and thus only occur in at most one of the fragmentation classes. Moreover, keep in mind that all masses are considered as SU-masses and are thus comparable. For a fragment $j\in M_{\mathrm{all}}$ , we define *m*_*j*_ as its mass. For a position *k* = 1,···,*S*, we introduce binary variables *y*_*j*,*k*_ to indicate whether fragment *j* covers position *k*. Analogously, we define *y*_*i*,*k*_ to indicate whether nucleotide *i* is assigned to position *k* in the sequence (uniqueness constrained by Eq. 7e). Under the previous assumption that all nucleotide masses are unique, let *n*_*i*_ be the mass of nucleotide *i*.

Using these variables, we define the optimization as a mixed-integer linear program (MILP) as in Algorithm 2: For each fragment, we determine the difference between its mass and the total mass of all nucleotides assigned to the positions covering it, and minimize over their sum (cf. Eq. 7a). We first assign each terminal fragment to start at its respective sequence end (cf. Eq. 7b, Eq. 7c); intact fragments cover the whole sequence (cf. Eq. 7d). Next, we ensure that each position is assigned exactly one nucleotide (Eq. 7e) and that all fragments are continuously aligned (Eq. 7f). Additionally, we enforce that the predicted sequence mass matches the intact mass within error range ϵ_intact_ (Eq. 7g, Eq. 7h). To circumvent the existence of a product and thus nonlinearity in the objective function, we introduce *z*_*i*,*j*,*k*_ as the product of *x*_*j*,*k*_ and *y*_*i*,*k*_, ensured by three additional constraints (Eqs. 7l–7n).

Using one of the existing linear optimization solvers like CBC or Gurobi (https://gurobi.com), we solve the optimization problem and select the assigned nucleotide for each position based on a cutoff of 0.9. We proceed analogously for the assignment of each fragment. If for any position no nucleotide can be assigned, we deem the whole prediction insufficient and report an empty sequence.

### LC-MS/MS Data Acquisition

End-labeled inosine RNA oligonucleotides (5′ 6-FAM tag and 3′ biotin TEG tag) are purchased from biomers.net GmbH. All other end-labeled oligonucleotides (5′ 6-FAM tag and 3′ biotin tag) are purchased from Integrated DNA Technologies Inc. 20–30 pmol of the synthetic end-labeled RNA oligonucleotide, were analyzed per injection in a Vanquish HPLC system connected to an Orbitrap Exploris 120 ESI-MS (Thermo Fisher Scientific Inc.). The sample components were separated with a flow rate of 0.2 mL/min in a solvent system of 50 mM HFIP (1,1,1,3,3,3-hexafluoro-2-propanol), 15 mM TEA (triethylamine), pH 9.00 (solvent A), and methanol (solvent B) at 80 °C on DNAPac RP 4 μm column (2.1 × 100 mm, Thermo Scientific). A multistep gradient (0% of solvent B in 0–2 min, followed by an increase from 0% to 3% until 5 min and 3% to 10% until 20 min, and from 10% to 20% until 25 min) was used for separation.

MS analysis was performed in the negative mode with a spray voltage of 2500 V, flow of sheath gas, aux gas, and sweep gas maintained at 35, 7, and 0, respectively, and ion transfer tube temperature and vaporizer temperature set at 300 °C and 275 °C, respectively. MS/MS analysis was performed using DDA with a scan range of 200–2000 *m*/*z*, with an MS1 resolution of 120,000, a normalized AGC of 200%, and an isolation window of 3 *m*/*z*. The normalized stepped collision energies used for each sequence are given in Table S1 of Supplementary E.

## Results

SpectrSeqTools is provided as a command-line application, enabling straightforward integration in automated workflows and execution in headless computing environments (cluster or cloud). It is installable via Bioconda^48^ and the source code is available under the GPL-3.0 license on GitHub: https://github.com/spectrseq/spectrseqtools. SpectrSeqTools is implemented in Python (https://python.org), using Polars (https://pola.rs) and NumPy^49^ for data handling, Altair^50^ for plotting, and Scikit-Learn^51^ for statistical functionality. It uses pulp (https://github.com/coin-or/pulp) for linear optimization, thereby supporting diverse solvers; in this work, we use the Gurobi solver (https://www.gurobi.com). SpectrSeqTools comes with a suite of tests for all major components that are continuously checked upon every commit via GitHub Actions, mitigating the risk of regressions in functionality, accuracy, and runtime performance.

We strove to evaluate the accuracy and runtime performance of SpectrSeqTools on experimental and simulated LC-MS/MS data. At the time of writing, no other software was available for comparison. The only related approach was RoboOligo, which, however, only claims to be able to sequence up to 12 nt (with decreased accuracy) and a subset of the known modifications.^39^ Moreover, the RoboOligo homepage was not reachable at the time of writing.

For reproducibility, the evaluation has been implemented as a Snakemake^52^ workflow that generates all shown results in a fully automatic way: https://github.com/spectrseq/spectrseqtools-evaluation. Further, the results can be interactively explored via a Snakemake report available online at https://github.com/spectrseq/spectrseqtools-evaluation-report.

### Sequence Prediction of RNA Oligonucleotides

We evaluated the sequencing algorithm, using 30 synthetic end-labeled RNA oligonucleotides (10-mers up to 30-mers) to represent a diversity of scenarios, ranging from unmodified sequences to those with single or double modifications. The test set included methylations on varying nucleotides (C, G, A, U), non-natural modifications (2′-fluoroA, 2′-MOE-rC), as well as inosine. Inosine was specifically included as a stress test for resolution, as it presents a significant challenge due to its nominal 1 Da mass difference from its corresponding unmodified nucleotide (adenosine). The experimental setup for the LC-MS/MS measurements is as provided in Section LC-MS/MS Data Acquisition. SpectrSeqTools reliably predicts oligonucleotide sequences up to 20-mers. The full list of considered oligonucleotide sequences is provided in the table in Supplementary E. The algorithm is also shown to correctly infer the sequence of oligonucleotides with the same nucleotide composition and intact sequence mass (Supplementary F) and custom non-natural modifications (Supplementary G), demonstrating its robustness.

In the following, we exemplarily discuss details for one of the more challenging sequences, 20mer-07. Figure 4a depicts the singletons identified during the preprocessing as well as their corresponding mass and nucleotide. Singletons and singleton identification are discussed further in Section Preprocessing. Note that for clarity, isobaric modifications are omitted. All six true nucleotides−A, C, G, U, Am, and Um−were detected; in addition, three false-positive singletons were identified (m^7^G, Ar­(p), and yW-58) but subsequently filtered out automatically during SpectrSeqTools’ prediction process. In Figure 4b and c, we portray the fragments used for the ladder building and subsequent inference as they appear in the MS spectra with their corresponding sequence and position as assigned by SpectrSeqTools. They represent the best fit for the mass of each fragment (given by right-side value) to minimize the difference to the mass determined by the predicted (given by the right-side value in parentheses). We observe that all fragments with higher errors (194, 214, 226) occur in the later steps of the 3′-ladder, indicating they were likely erroneously classified as 3′-fragments. We assume that this is due to the lack of sufficient observed fragment masses in the raw data that would preserve the remaining steps of the 3′-ladder. Similarly, internal fragments with high errors may also have been erroneously classified as internal (68, 202). However, this did not hamper the successful inference of the correct final sequence, also due to the fact that SpectrSeqTools additionally considers internal fragments in its joint sequence inference and fragment alignment (cf. Algorithm 2).

### Computational Efficiency

SpectrSeqTools is designed to be applicable on any personal laptop; we used a Lenovo ThinkPad X1 Carbon with 1 core and up to 5 GB of memory. Depending on the sequence complexity and number of raw MS/MS scans, a prediction of sequences ≤30 nt takes up to 5 min on such a machine.

For time-efficient parallel evaluation, we ran the below simulations on the de.NBI cloud with AMD Epyc (28 cores, 2.3 Ghz) and 64 GB RAM. Exemplarily, we measured the performance of the simulation of different sequence replicates (cf. Section Robustness of SpectrSeqTools on Simulated Data) with Snakemake’s benchmark function. Figure 4b and Figure 4c plot the number of fragments against the runtime and memory for the simulated (gray) and experimental (red) data, respectively. Note that memory values below 5 MB and missing values were considered artifacts caused by lazy memory allocation and discrete sampling, and thus filtered out.

**Figure 5:**
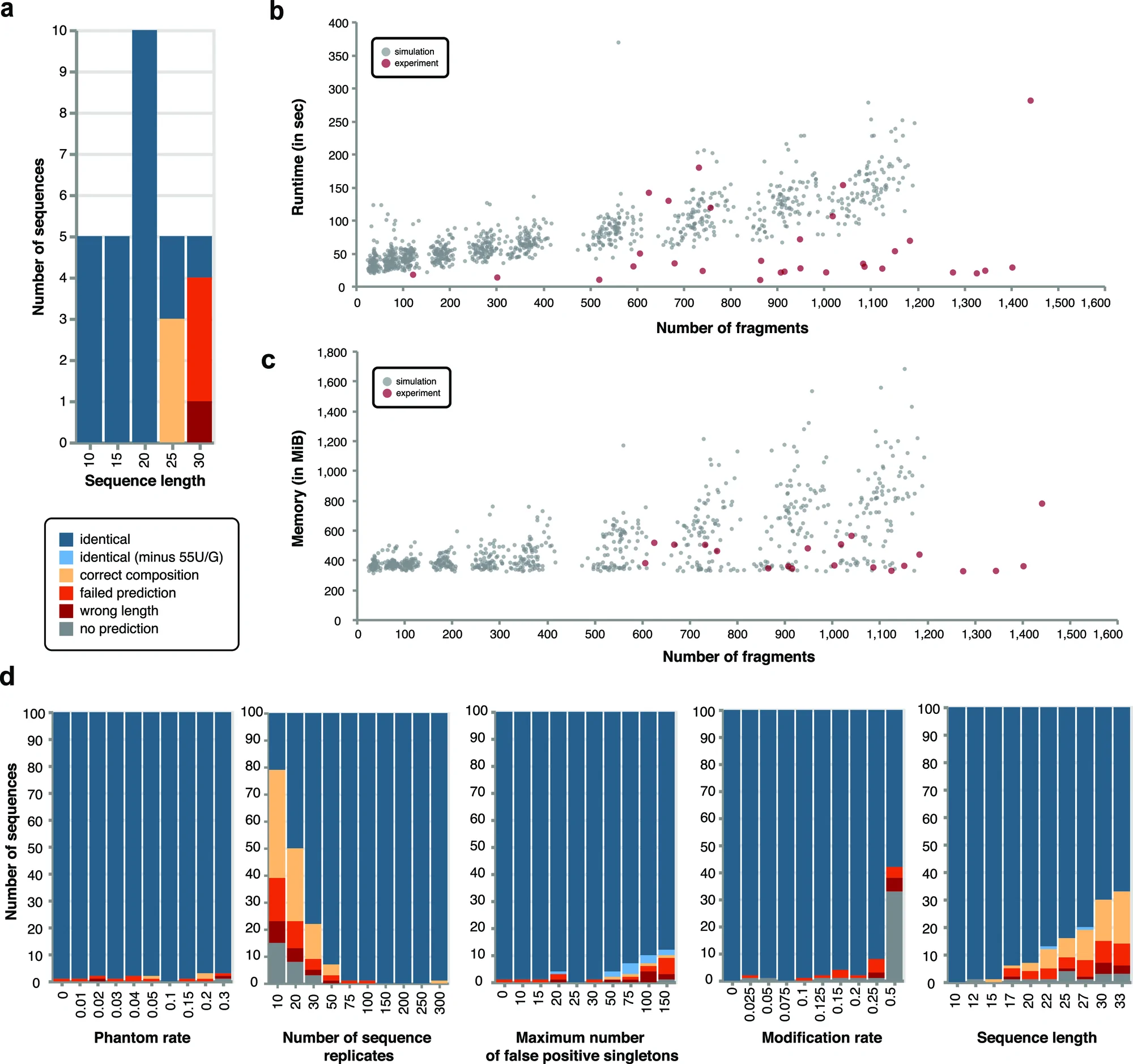
Experimental and simulated results. **a:** Overview of prediction results for all sequences used for analysis separated by sequence length. **b:** Number of fragments vs. runtime of the 30 experimental samples (red) and 1000 simulated runs (gray). **c:** Number of fragments vs. memory usage of 30 experimental samples (red) and 1000 simulated runs (gray) **d:** Prediction results for stratified simulation over different parameters. Default settings are a phantom rate of 0%, 100 replicates per sequence, a maximum of 10 false positive singletons, a modification rate of 10%, and a randomly selected sequence length between 10 and 20 nt. For the simulations, all parameters are set to their default value except the one being evaluated. The results are divided into six categories: fully correct prediction (″identical″), correct except switches between G and 55U, which are within the allowed tolerance τ_prediction_ (″identical (minus 55U/G)″), correct composition in the wrong order (″correct composition″), correct length but wrong composition (″failed prediction″), wrong selected length (″wrong length″), and an empty prediction due to insufficient evidence to either build a full sequence from the two ladders or resolve at least one position within the given time frame of the linear optimization (″no prediction″).

We observe that for larger numbers of fragments, the variability of both runtime and memory increases as well. The runtime of the experimental data falls into similar ranges as the simulations. For up to 20-mers, it is even consistently <2 min and falls below the minimum of the simulated runs. This could be due to the fewer considered modifications in comparison to both the longer experimental sequences and the simulations. Simulated and experimental data show similar trends in memory consumption, with a consistent minimum of ca. 300 MiB (due to the size of the traceback matrix).

### Robustness of SpectrSeqTools on Simulated Data

In addition to the evaluation of the prediction performance on real LC-MS/MS measurements (synthetic end-labeled oligonucleotides), we simulated fragment observations of random sequences with varying lengths in order to assess the prediction performance of SpectrSeqTools on a larger scale while varying data quality and complexity. We generated sequences of a random length between 10 and 20 bases (uniform distribution). Each sequence was drawn from the full nucleotide alphabet (cf. Supplementary D), whereby the likelihood of selecting a modified base was given by a modification rate μ (and vice versa for an unmodified base by 1 – μ). For each sequence, we created a certain number of replicates (i.e. copies of the same sequence) and simulated fragmentation independently on each replicate, by first selecting the number of fragmentation sites and then their positions. For the former, we used a geometric distribution with *p* = 0.3, because this most closely resembles the distributions found in the experimental data (cf. Supplementary H). For the latter, we chose to use a beta distribution with *a* = *b* = 2. The hill shape imitates a uniform distribution of fragmentation sites across the sequence length. However, the reduced rates toward the sequence ends avoid an excess of terminal fragments compared to internal fragments. After the fragmentation, we determined the corresponding fragment and its mass (assuming c/y-fragmentation) for all neighboring sites and added random noise uniformly drawn from [−5,5] ppm. In addition, we introduced a so-called *phantom rate* that defines the chance of encountering a *phantom fragment*, which represents deisotoped and deconvoluted noise peaks from the raw spectra. Simulated fragments were randomly considered as a phantom one according to this rate and distorted via a uniformly drawn random mass within [−1,1] kDa.

Since the above simulation procedure directly generates masses, not spectra, we generated artificial singleton observations (i.e. single nucleotides usually observed from spectra, used by SpectrSeqTools to determine the required nucleotide alphabet; cf. Section Preprocessing). For this, we first chose all nucleotides that appear in the given sequence, and then filled up to a given maximum number (mimicking noisy additional singleton observations). If the number of true nucleotides exceeded the given maximum number of singletons, we still select all of the former regardless of the latter.

In Figure 5d, we show the robustness of SpectrSeqTools based on this simulated data. For each comparison, we set all parameters to their default values and only adjusted the one currently evaluated.

We observe that changing the phantom rate barely affects the accuracy, so that even for a rate of 30%, we still predict >95% of sequences correctly. This shows that our method is robust against noise peaks and can handle even very noisy data.

Looking at the number of sequence replicates, the comparison shows that for few replicates (around 75), SpectrSeqTools is already highly accurate. This indicates that the software does not require much data to allow accurate sequence reconstruction.

The third plot validates the usage of the singleton identification: For the synthetic oligonucleotides, the expected number of singletons is around 10, for which SpectrSeqTools has nearly 100% accuracy. With an increasing number of false-positive singletons, the accuracy is reduced, but even for the full alphabet of around 144 nucleotides, >85% of the sequences could still be reconstructed.

The fourth barplot shows that SpectrSeqTools is largely independent of the number of modified bases in the sequence; only for an unrealistically high modification rate of 50% the accuracy falls below 90%.

Lastly, we show that while the accuracy decreases with increasing sequence length, SpectrSeqTools maintains a high accuracy of over 90–95% for up to 20-mers. Errors mostly stem either from unresolved positions due to insufficient fragmentation or incorrect assignment of rare modifications, which can both be credited to a simulation setup that is designed to unrealistically favor such cases.

## Conclusion

Post-transcriptional RNA modifications play essential roles in regulating fundamental cellular processes, yet their precise biological functions remain poorly understood. A major obstacle to advancing this field is the lack of reliable high-throughput methods for comprehensive analysis. Because most RNA modifications are difficult to detect using existing sequencing technologies, our knowledge of their abundance, distribution, and functional implications remains largely incomplete.

Mass-spectrometry-based de novo RNA sequencing, including the parallel detection of post-transcriptional modifications, offers a promising route to overcome these limitations. However, previously published approaches have been constrained either by very short read lengths, a limited set of detectable modifications, or reliance on manual, non-automated workflows. Here, we introduce SpectrSeqTools, a novel fully automated computational framework for inferring RNA sequences together with their modification profiles from MS/MS data.

SpectrSeqTools incorporates four key algorithmic innovations that substantially improve the efficiency of MS/MS-based RNA sequencing: normalization of fragment masses into standard units, the consideration of singleton fragments for reducing the nucleotide alphabet search space, an implicit representation of the sequence composition space using a traceback matrix, and joint sequence inference and fragment alignment formulated as a linear optimization problem. To our knowledge, the latter is the first method that is able to consider internal fragments at all, thereby greatly increasing the available evidence and thus improving both accuracy and robustness. The former three dramatically increase the computational feasibility, bringing the typical lengths of small biological RNAs in reach. Together, these advances enable comprehensive consideration of known RNA modifications and allow accurate sequencing of end-labeled RNA molecules up to 20 nt in length, as demonstrated by our experimental data. Moreover, analyses of simulated data show that SpectrSeqTools remains robust and accurate even under increasing levels of artificial noise. Importantly, the analysis is not limited to small RNAs, but could−in principle−also be extended to longer RNAs following RNase digestion.

While SpectrSeqTools currently supports by default 144 naturally occurring RNA modifications (cf. Supplementary D), some of them are isobaric and thus generally indistinguishable without prior chemical labeling (e.g., CMC derivatization,^53^ cyanoethylation,^54^ and bisulfite labeling^55^ for pseudouridine detection). However, SpectrSeqTools supports the usage of custom alphabets and can thus readily be extended to include synthetic, user-defined modifications. Besides automated analysis of biologically derived RNA, this flexibility makes the platform well-suited also for quality control of synthetic oligonucleotides for therapeutic or diagnostic applications. Notably, the achieved length of up to 20 nt already falls within the size range of small regulatory RNAs or therapeutic oligonucleotides, underscoring the relevance and practical utility of our approach.

To further increase read length, one promising direction is to reformulate the skeleton construction as a graph-based problem, enabling explicit modeling of increasing uncertainty at later reconstruction stages by representing alternative fragment ladders as graph traversals. In addition, we will introduce probabilistic Bayesian inference that incorporates prior modification frequencies and reports uncertainty via position-specific posterior nucleotide distributions. This approach will naturally allow the simultaneous consideration of all fragmentation types while weighting them according to their prior probabilities. In scenarios where candidate sequences are known a priori, we will enable their use to constrain the search space of SpectrSeqTools across all major stages of the pipeline, including composition inference, skeleton construction, and sequence reconstruction. This strategy is expected to further improve both accuracy and computational efficiency, thereby enabling the application to longer RNA species such as tRNAs, whose primary sequences are readily available from genomic annotations.

In summary, SpectrSeqTools enables fully automated, true de novo reconstruction of RNA sequences, including comprehensive post-transcriptional modification profiling, directly from LC-MS/MS raw data. By supporting 144 RNA modifications by default (and further extension via custom alphabets), the platform substantially expands current analytical capabilities and enables systematic, unbiased interrogation of RNA modification complexity. As a rapid and cost-effective orthogonal approach to traditional sequencing methods, SpectrSeqTools broadens the applicability of nucleic acid mass spectrometry and provides a robust framework for decoding dynamic RNA modification patterns.

## Supplementary Material



## Data Availability

The raw data are available on Zenodo: 10.5281/zenodo.18457758.

## Notes

Filters used in SpectrSeqTools; Deconvolution; Effect of collision energy on singleton detection; Distribution of monoisotopic masses of modified and unmodified nucleosides; List of oligonucleotide sequences; Isobaric oligonucleotides; Non-natural modifications; Comparing simulated and experimental fragment mass distributions (PDF)
