# Supplementary material for: SpectrSeqTools: LC-MS/MS-Based De Novo Sequencing of Small Ribonucleic Acids with Post-Transcriptional Modifications
Source: Anal Chem. 2026 Sep 25;98(39):28784–97. doi: 10.1021/acs.analchem.6c02546 (PMC13647978; doi:10.1021/acs.analchem.6c02546)
Supplement: Supplementary file 1 [file ac6c02546_si_001.pdf]

Supplementary Information

SpectrSeqTools: LC-MS/MS-Based De Novo Sequencing of Small Ribonucleic Acids with Post-Transcriptional Modifications

Laura C. Kühle[1,3,#], Moshir Harsh[1,3,#], Shanice Jessica Hermon[1,2,#], Juan Magalang[1,2], Johannes Köster[3,4,*], Sidney Becker[1,2,*]

[1]Max Planck Institute of Molecular Physiology, Otto-Hahn-Straße 11, 44227, Dortmund, Germany
[2]Department of Chemistry and Chemical Biology, Technical University Dortmund Otto-Hahn-Straße 6, 44227, Dortmund, Germany
[3]Bioinformatics and Computational Oncology, Institute for AI in Medicine (IKIM), University Hospital Essen, Faculty of Medicine, University of Duisburg-Essen, Girardetstraße 2, 45131, Essen, Germany
[4]German Cancer Consortium (DKTK), University Hospital Essen, Hufelandstraße 55, 45147, Essen, Germany
[#]These authors contributed equally to this work.
[*]Corresponding authors: johannes.koester@uni-due.de, sidney.becker@mpi-dortmund.mpg.de

Contents

A Filters in SpectrSeqTools **S3**
A.1 Filters after Stage 1 (Preprocessing) S3
A.2 Filters after Stage 2 (Fragment classification) S3
A.3 Filters after Stage 3 (Skeleton building) S3

B Deconvolution **S5**

C Effect of collision energy on singleton detection **S6**

D Distribution of monoisotopic masses of modified and unmodified nucleosides **S7**

E List of oligonucleotide sequences **S8**

F Isobaric oligonucleotides **S9**

G Non-natural modifications **S11**

H Comparing simulated and experimental fragment mass distributions **S12**

Suppl. A Filters in SpectrSeqTools

To compensate for the abundance of fragments created by the conservative fragment classification approach as well as the possibly extensive nucleotide alphabet, we apply a variety of filters to reduce the considered amount between the major stages of SpectrSeqTools. In the following, we provide a list explaining the function of each filter in order of application in SpectrSeqTools.

A.1 Filters after Stage 1 (Preprocessing)

Filter nucleotide alphabet by singletons
If SpectrSeqTools is provided raw spectra and can infer singleton peaks, it uses those to reduce the considered nucleotide alphabet $\mathcal{N}$, assuming that each nucleotide in the measured molecule will also occur as a singleton peak. Canonical bases are thereby always kept in the alphabet, even if not detected as a singleton.

Filter fragments by intensity
To avoid using low-quality fragments, an intensity cutoff can be provided, which determines the minimum intensity required to be considered for the prediction. By default, this value is set to the 80th percentile of the distribution of intensities of the fragments.
Note that we lowered this value for 20mer-05 (40th percentile; due to contamination) and 20mer-10 (65th percentile).

A.2 Filters after Stage 2 (Fragment classification)

Filter fragments by intact mass
After the mass standardization (SU) and thus the related duplication of original fragments, we apply two initial fragment filters to remove already infeasible masses. The first filter compares the fragment masses to the intact mass m_{intact} and removes all intact fragments that do not match it (within error range ϵ_{intact}) as well as all fragments of any other class that exceed $m_{\text{intact}} + \epsilon_{\text{intact}}$.

Filter fragments with the traceback matrix
The second initial filter aims to remove fragments whose SU-mass m cannot be explained with the current nucleotide alphabet (i.e. has no valid composition). The filter verifies whether the corresponding entry in the last row of the DP for any integer in the range $[m - \tau_{\text{prediction}} \cdot m, m + \tau_{\text{prediction}} \cdot m]$ is reachable and removes the fragment if this is not the case, with $\tau_{\text{prediction}}$ being the tolerance parameter as defined before. Additionally, this filter is applied each time the nucleotide alphabet is reduced.

Filter fragments and nucleotide alphabet by compositions
To counterbalance a possible liberal singleton identification, an additional filter recursively reduces both the nucleotide alphabet as well as the considered fragments based on compositions computed over the difference between successive mass-ordered terminal fragments (similar to the approach for skeleton building in Section Skeleton building), combined with a sliding window to restrict the maximum difference. Only nucleotides appearing in at least one of the yielded compositions are retained. If that reduces the alphabet, the set of fragments is filtered with the above traceback matrix filter, and the set of compositions is recomputed. The process is repeated until the alphabet is no longer reduced.

A.3 Filters after Stage 3 (Skeleton building)

Filter internal fragments based on skeleton
Assuming the skeleton sequence is correct, we discard for all used terminal fragments their alternative interpretations (cf. Section Fragment classification). Note that intact fragments are considered as both 5'- and 3'-fragments during the ladder building, hence we arbitrarily remove them in the set of 3'-fragments to avoid counting them twice in later stages.

Filter nucleotide alphabet based on skeleton

Analogously to the singleton filter above, we remove all nucleotides from the alphabet that are not found in any position of the skeleton sequence.

Filter fragments with linear optimization

The measurement process can introduce random noise peaks, subsequently leading to phantom fragments that do not fit with the rest of the data. Such fragments can be removed once the skeleton sequence has been determined. For this, we utilize the linear optimization approach, restricted to one fragment (with mass m) at a time (Section Sequence inference), thereby trying to align it against the provided skeleton sequence. If the error of the result exceeds $\tau_{\text{prediction}} \cdot m$ or no solution is found within a given number of seconds (5 by default), we consider m to be corresponding to a phantom fragment and reject it. Note that – because we only consider c/y-fragmentation – experimental data with any other fragmentation is also treated as a phantom fragment and rejected.

Suppl. B Deconvolution

Molecules that are analyzed with mass spectrometry produce spectra on an MS/MS scan, which presents as a distribution of intensity peaks as a function of m/z. These MS/MS scans originate from peaks on a precursor MS scan, selected by the LC-MS. Each peak on this distribution (also called isotopomer envelope) corresponds to an isotopic variant of the molecule, which occurs as a consequence of natural abundances of stable isotopes.[1] For complex molecules such as RNA, the first (thus lightest) peak of this envelope is defined as the monoisotopic peak, representing the mass of the molecule when it is composed entirely of the most abundant isotope of each atom. Deisotoping is the process of inferring the monoisotopic peak from each isotopomer envelope. Isotopomer envelopes can be detected by fitting theoretical isotope distributions on the measured peaks.[2] Monoisotopic peaks that originate from the same molecule may still be separated by m/z, since they carry different charge states. Deconvolution aggregates these similar peaks together by recomputing the corresponding neutral mass.

In SpectrSeqTools, we utilize the `ms_deisotope` library[3] to perform automatic deisotoping by detecting these isotopomer envelopes and choosing the monoisotopic peak. We configure the *averagine model*[4,5] to use $C_{9.5}H_{12.75}N_{3.75}O_7P_1$ as an average of all four unmodified bases since these are the most common bases found in an RNA sequence, with distribution truncation at the 90th percentile. Further, we set the minimum threshold for Liu's scoring function[2] to 150. We note that only MS/MS scans with a precursor MS charge state greater than a given value are selected for deisotoping. This minimum precursor charge state is set to 3 by default, as it has been observed that there is a higher likelihood that peaks with a precursor charge lower than this value come from noise. The maximum charge state considered is taken from the charge state of the precursor peak if it is available; otherwise, the maximum charge state is 30, to detect closely-spaced isotope distributions.

After deisotoping the MS/MS scans, `ms_deisotope` deconvolutes the monoisotopic peaks per scan to obtain neutral masses. Neutral masses from all MS/MS scans are collected in one set. To account for measurement noise, neutral masses within a mass error threshold (10 ppm by default) are averaged together, and their intensities are summed (cf. Figure 4b).

The corresponding MS precursor peaks of each MS/MS scan are also deisotoped and deconvoluted similarly. However, since MS precursor scans form less noisy isotopomer envelopes compared to MS/MS scans, we use the same scoring function by Liu, but weighted by a likelihood ratio that the experimental peaks in the isotopomer envelopes match with the theoretical averagine peaks. This scoring function is given as an option in `ms_deisotope`. We use the default minimum scoring function of 10. The precursor neutral mass with the highest intensity is used as the intact sequence mass, given that this mass is greater than the sum of the masses of the end tags as defined in Section Algorithmic innovations.

Suppl. C Effect of collision energy on singleton detection

Orbitrap MS instruments, such as the instrument used to collect the LC-MS/MS data in this text, utilize high-energy collision dissociation (HCD) to break ions and produce fragments to be analyzed in the mass spectrometer. This experimental parameter is inversely proportional to the size of the MS/MS fragments generated, as higher energies would result in shorter fragments and vice versa.

As such, the collision energy may affect the sequence prediction, especially the singleton identification, since it requires that the MS/MS fragment of a single nucleotide is detected while still being able to detect MS/MS fragments of varying lengths. To diversify the lengths of fragments that are to be analyzed, step-wise collision energies (given in percentage triples) are used to produce a more comprehensive mass spectrum. We investigate the effect of varying the step-wise collision energies on the performance of the singleton identification.

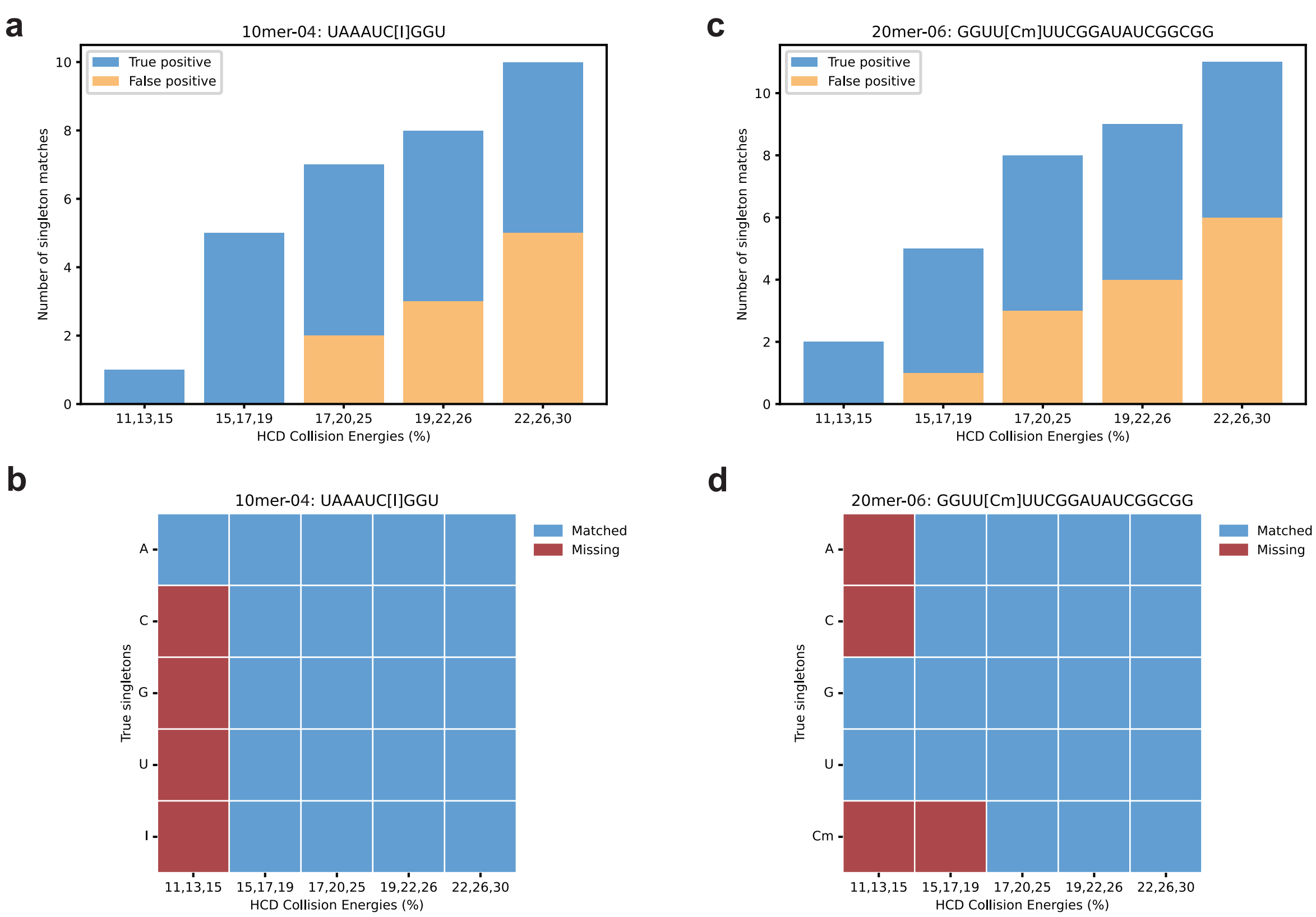

Figure S1: Singleton identification results as a function of step-wise collision energies for two samples enumerated in Figure 4: 10mer-04 (a,b) and 20mer-06 (c,d). **a,c:** Number of singletons detected, aggregated by true positive matches and false positive matches. **b,d:** Comparing detected singletons to the unique bases found in the reference sequence.

Figure S1a,c compares the singletons that exist in the reference sequence (i.e. true positive matches) and singletons that do not (i.e. false positive matches) as a function of the step-wise collision energies. Lower step-wise energies reduce the number of false positives, but also reduce the overall count of singletons that were matched. This implies that singletons that should be in the sequence were not detected, i.e. false negative matches. Figure S1b,d illustrates the existence of false negative matches at low step-wise energy values.

Suppl. D Distribution of monoisotopic masses of modified and unmodified nucleosides

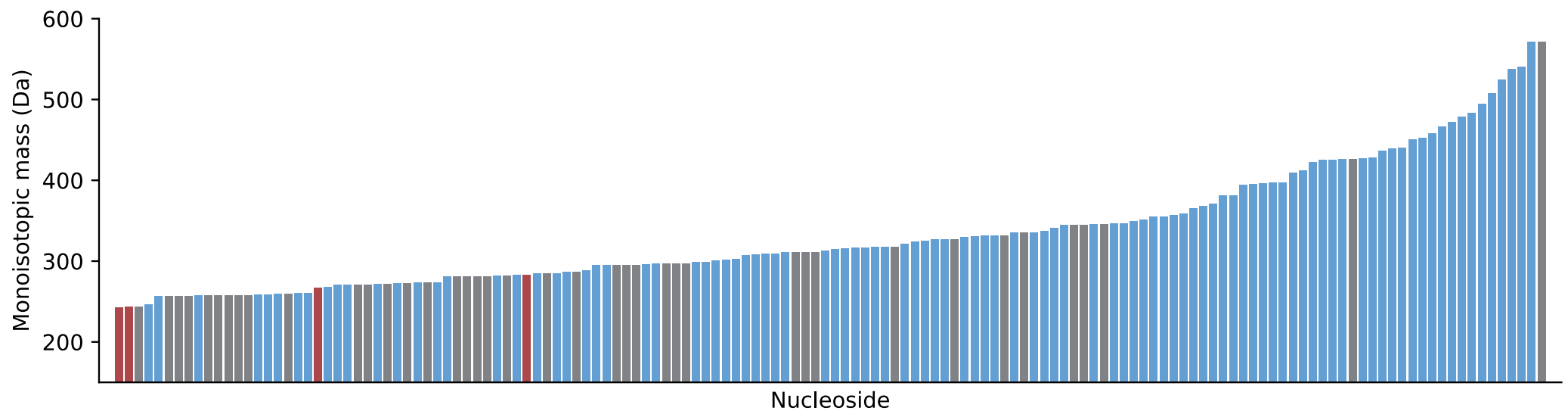

Figure S2: Distribution of monoisotopic masses of modified or unmodified nucleosides, sorted from lightest to heaviest. Unmodified nucleosides are marked in red (4 nucleosides), modified nucleosides are marked in blue (100 nucleosides). Modifications marked in gray (40 nucleosides) are isobaric, i.e. have the same mass as their representative one. SpectrSeqTools outputs the representative along with the isobaric alternatives in the predicted sequence.

Figure S2 shows the distribution of monoisotopic masses of modified and unmodified nucleosides found in MODOMICS. There are 23 groups of nucleosides that have the same monoisotopic mass; these are grouped together in the nucleoside alphabet that is used in processing.

Suppl. E List of oligonucleotide sequences

Table S1 shows the list of the 30 oligonucleotide sequences used for the evaluation of SpectrSeqTools. This demonstrates the capability of the software to infer the correct sequences and with modified nucleotides reliably up to a 20-mer sequence. The step-wise collision energies for each sample are also provided; this is discussed further in Supplementary C.

Table S1: List of oligonucleotide sequences used with SpectrSeqTools. Status ● refers to a correctly inferred sequence, ● refers to a correct composition but incorrect order, ● refers to a failed prediction, ● refers to a wrongly-estimated sequence length.

Sample name	Sequence	Status	Step-wise collision energies (%)
10mer-01	GAUUGUCGUG	●	17, 20, 25
10mer-02	GAU**[Cm]**UAGAUC	●	17, 20, 25
10mer-03	UUAGCCCG**[Gm]**A	●	17, 20, 25
10mer-04	UAAAUC**[I]**GGU	●	17, 20, 25
10mer-05	UCGA**[I]**UUGAG	●	17, 20, 25
15mer-01	UAGCGUUUGAUGAUC	●	15, 17, 19
15mer-02	GCCGCCUGAUGAGCG	●	15, 17, 19
15mer-03	AAGAGACCUAAAGCC	●	15, 17, 19
15mer-04	UAAAGUACAUUAACA	●	15, 17, 19
15mer-05	UGCCAUUAUACAUGA	●	15, 17, 19
20mer-01	CAACAAGCGAGAGGUUCGAA	●	17, 20, 25
20mer-02	AGAGGCUAAUAGUAAGUUAA	●	17, 20, 25
20mer-03 (isobaric_01)	CCGAUGUGGGGGGUAUCUCG	●	15, 17, 19
20mer-04 (isobaric_02)	CCUGGCUCGAUGAGGUGUGG	●	17, 20, 25
20mer-05 (isobaric_03)	GUAGGCGUGAUGCCCGUGUG	●	17, 20, 25
20mer-06	GGUU**[Cm]**UUCGGAUAUCGGCGG	●	17, 20, 25
20mer-07	CCA**[Am]**GUGCUAA**[Um]**AAAUUGCC	●	17, 20, 25
20mer-08	UGCCUCACCAC**[I]**UCAUCCUG	●	17, 20, 25
20mer-09 (2MOErC)	AACGAUGUAU**[2MOErC]**GGGACGUGA	●	17, 20, 25
20mer-10 (2FluoroA)	UCCCGAACGUCCACCA**[2FluoroA]**UUA	●	17, 20, 25
25mer-01	AUAGAUAGCCUAAAUCUCAGAGCUA	●	17, 20, 25
25mer-02	CAGUAUGGUAGAUAGAACAACGAAG	●	17, 20, 25
25mer-03	AGGGCCAAUGCUGAAAACUAGAUAG	●	17, 20, 25
25mer-04	CUCAGUCGGCUAUCUACCCUAACCC	●	17, 20, 25
25mer-05	CUCAGCUUCAAGAACGACCUCGAAU	●	17, 20, 25
30mer-01	CAGCCCACCACGUGUAGUGGGCCUAGCAGU	●	17, 20, 25
30mer-02	CUCCACGAUUAGCACUAUUGCCUGCGGGAU	●	17, 20, 25
30mer-03	AAACUAGUAAGUGGUUGGAAACAACUCGUA	●	17, 20, 25
30mer-04	AGGUCGGAAGGCUGGGUCCUGGCCUCAAAU	●	17, 20, 25
30mer-05	ACUCUACAGGACCGCGUGGCCCUUUAGCAG	●	17, 20, 25

Suppl. F Isobaric oligonucleotides

In this section, we demonstrate the robustness of SpectrSeqTools by sequencing isobaric oligonucleotides. The sequences have identical nucleotide compositions and intact sequence masses, making them indistinguishable based on the intact sequence mass alone. Figure S3, Figure S4, and Figure S5 show the SpectrSeqTools output of three isobaric sequences, similar to the output from Figure 4 of the main text. Indeed, SpectrSeqTools correctly reconstructs all three sequences, indicating that the software can distinguish isobaric oligonucleotides, demonstrating that the sequence assignment is from MS/MS fragmentation that is specific to each sequence.

We also note that in Figure S5, the unmodified base A was not found in the singleton scans, but the correct sequence was still inferred since it is imposed that all four unmodified bases are included in the alphabet regardless of whether or not it is found as a singleton in the MS/MS scans.

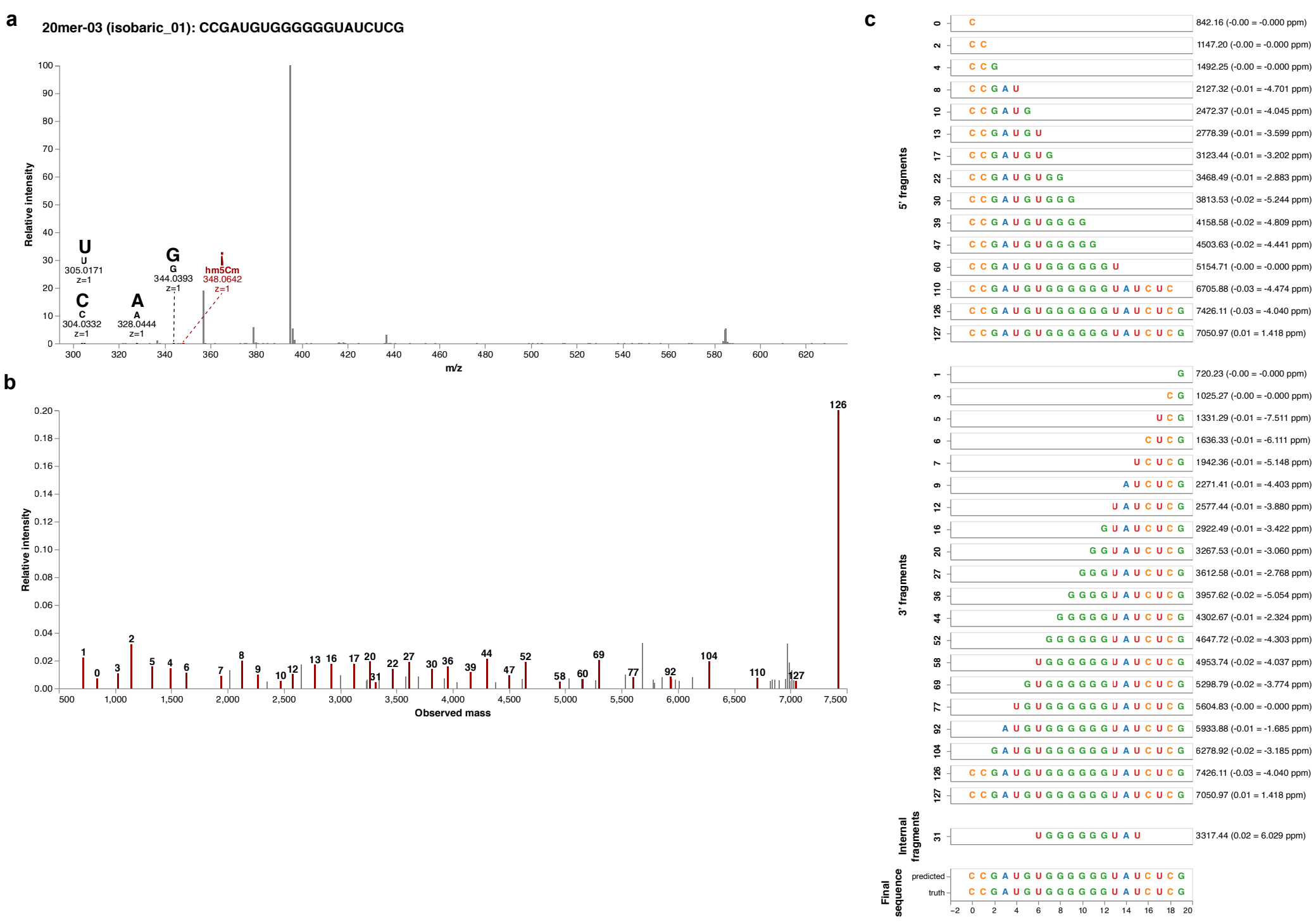

Figure S3: SpectrSeqTools output for an oligonucleotide that is isobaric to Figure S4 and Figure S5. **a:** Sample LC-MS/MS spectra with the identified singletons marked. **b:** Deconvoluted and deisotoped data with the identified fragments marked red. **c:** Sequence assignment map from SpectrSeqTools.

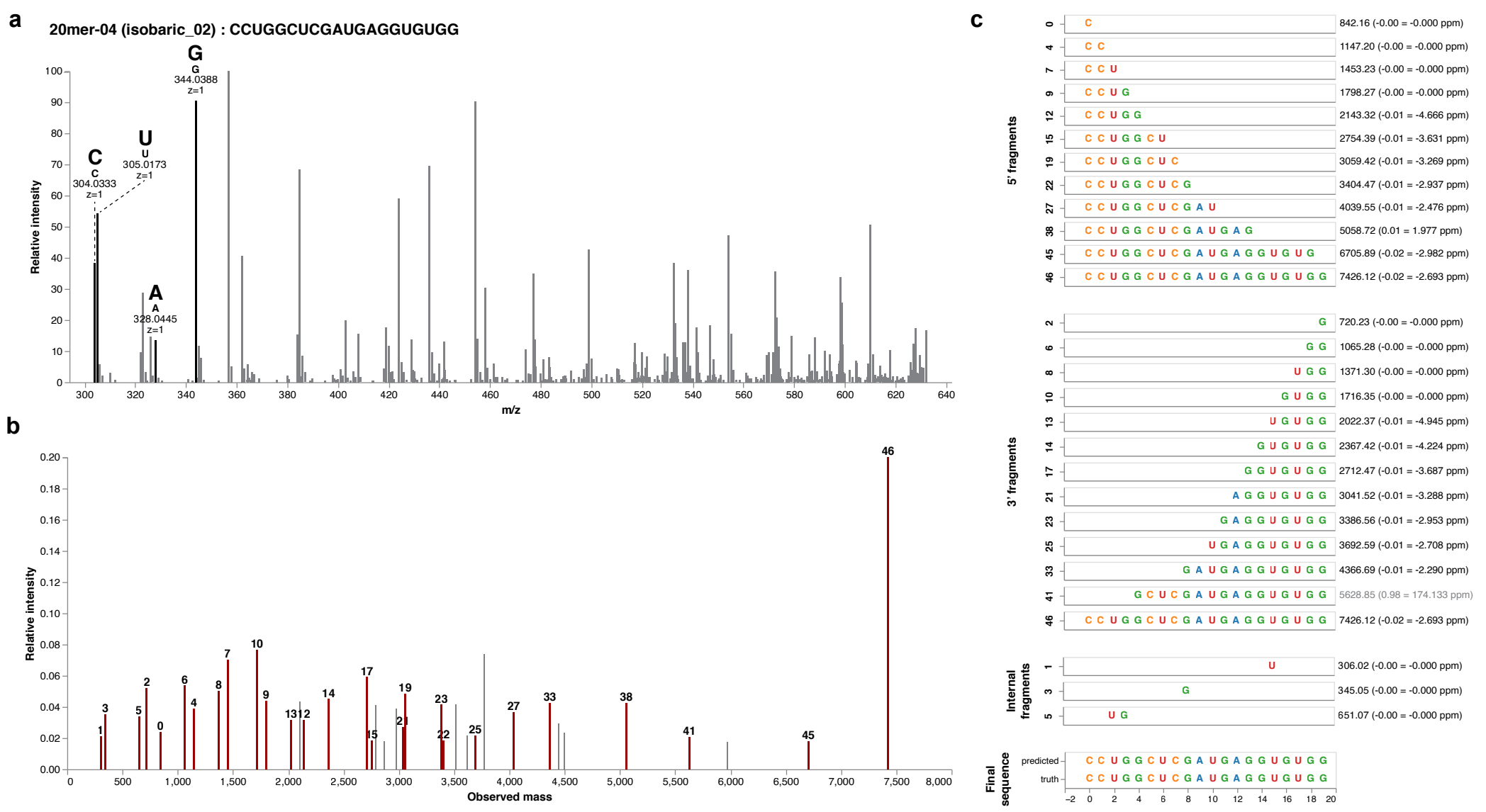

Figure S4: SpectrSeqTools output for an oligonucleotide that is isobaric to Figure S3 and Figure S5. **a:** Sample LC-MS/MS spectra with the identified singletons marked. **b:** Deconvoluted and deisotoped data with the identified fragments marked red. **c:** Sequence assignment map from SpectrSeqTools.

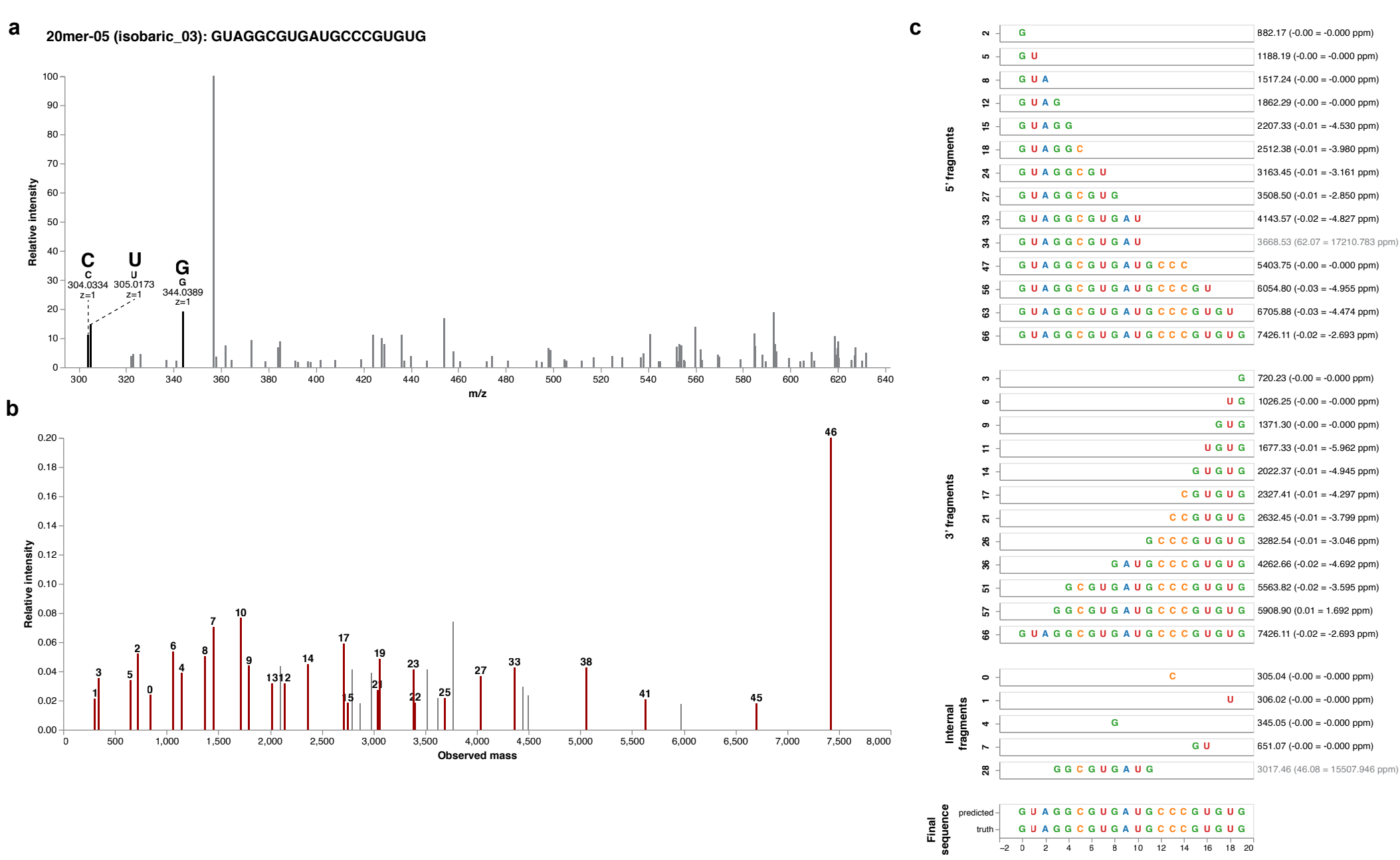

Figure S5: SpectrSeqTools output for an oligonucleotide that is isobaric to Figure S3 and Figure S4. **a:** Sample LC-MS/MS spectra with the identified singletons marked. **b:** Deconvoluted and deisotoped data with the identified fragments marked red. **c:** Sequence assignment map from SpectrSeqTools.

Suppl. G Non-natural modifications

In this section, we sequence modified oligonucleotides with SpectrSeqTools. The modifications are non-natural and not present in MODOMICS,[6] therefore they were defined as custom modifications for the analysis. Figure S6, and Figure S7 show the SpectrSeqTools output of these modified sequences, similar to the output from Figure 4 of the main text. SpectrSeqTools correctly reconstructs both sequences and identifies the corresponding modifications as singletons.

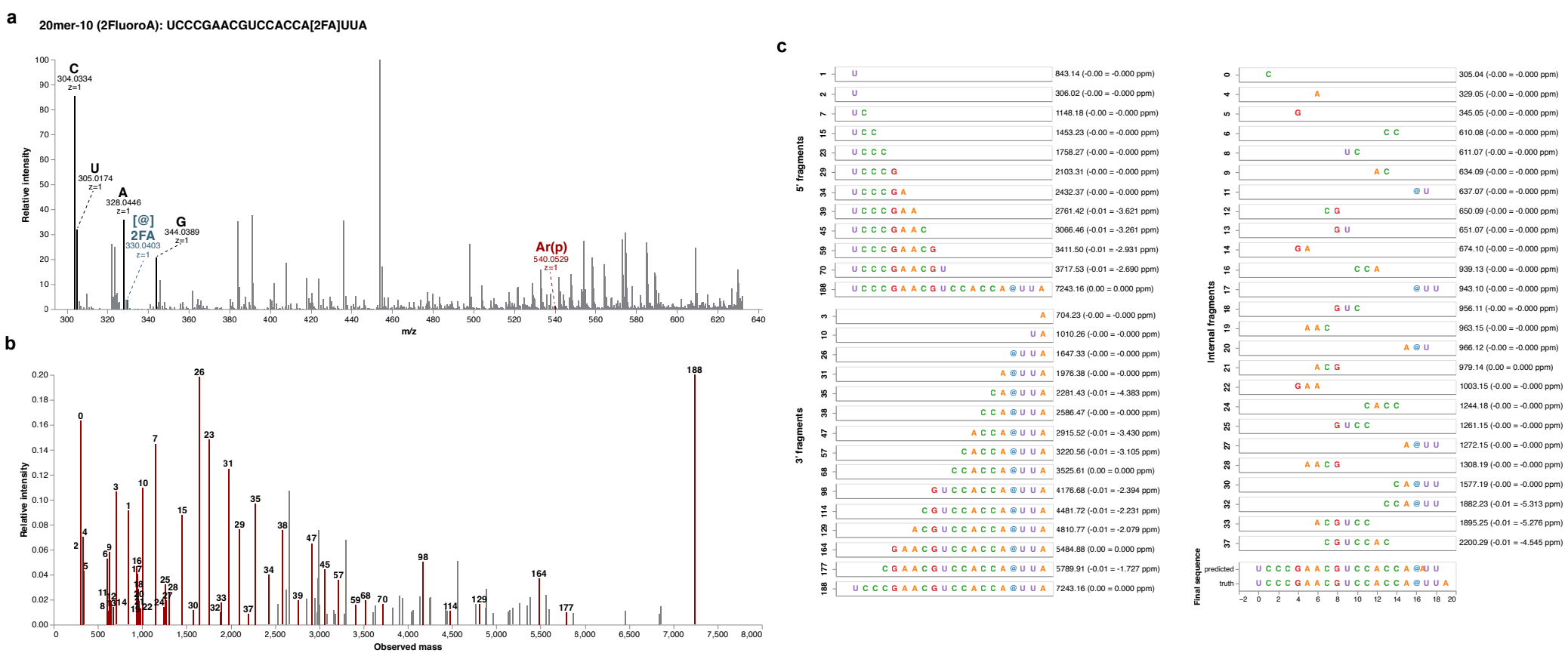

Figure S6: SpectrSeqTools output for the oligonucleotide with the modification 2-fluoroadenine (2FluoroA). Sample LC-MS/MS spectra with the identified singletons marked. **b:** Deconvoluted and deisotoped data with the identified fragments marked red. **c:** Sequence assignment map from SpectrSeqTools.

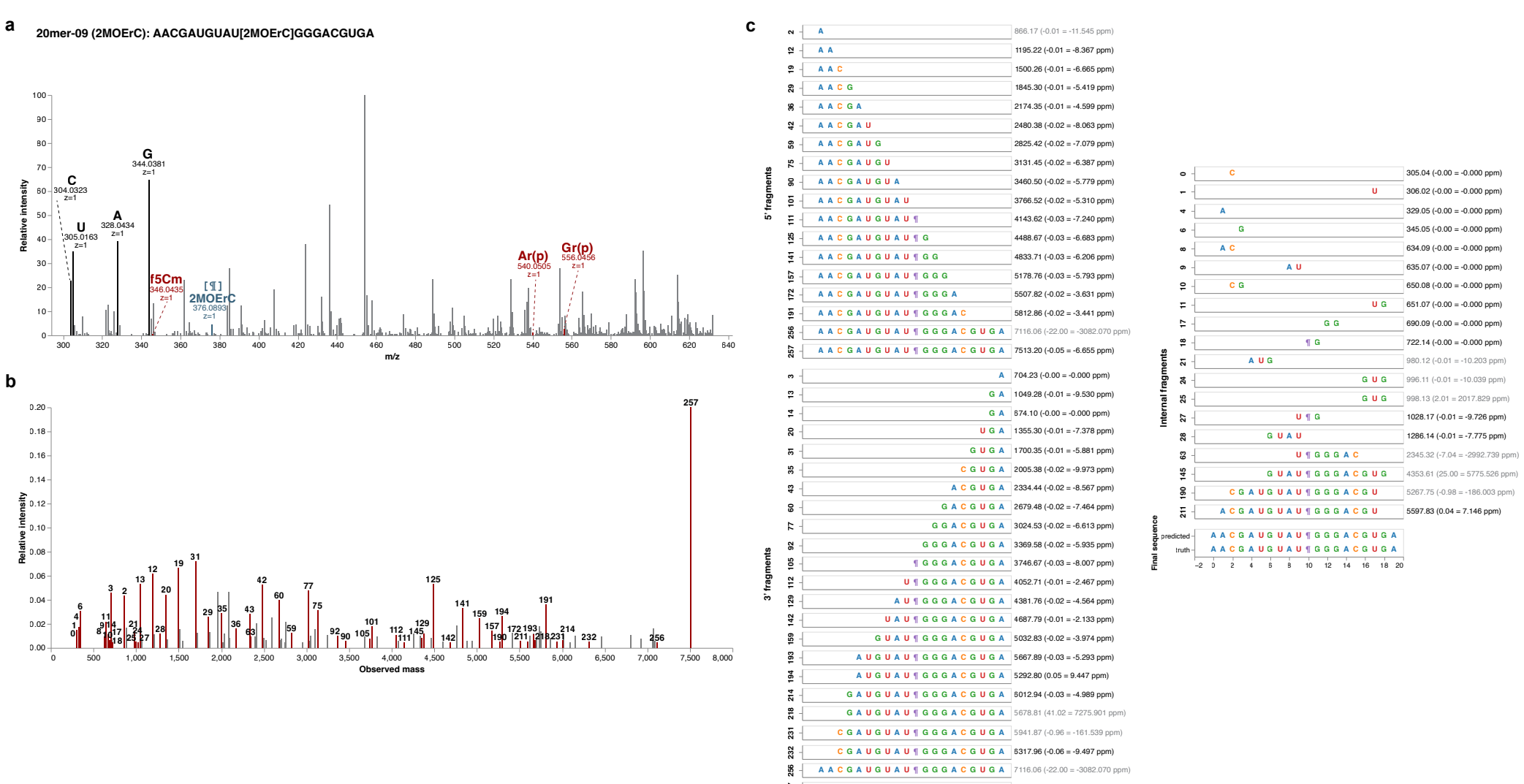

Figure S7: SpectrSeqTools output for the oligonucleotide with the modification 2'-O-Methoxyethyl-5-methylcytidine (2MOErC). **a:** Sample LC-MS/MS spectra with the identified singletons marked. **b:** Deconvoluted and deisotoped data with the identified fragments marked red. **c:** Sequence assignment map from SpectrSeqTools.

Suppl. H Comparing simulated and experimental fragment mass distributions

Section Robustness of SpectrSeqTools on simulated data outlines the details of the simulations that were used to generate Figure 5, where the simulated fragment lengths were drawn from a geometric distribution with $p = 0.3$. We compare the distributions of the simulated fragments with the actual observed fragments to see the justification of the decision. Let L be a simulated fragment length, i.e. $L \sim \text{Geom}(p)$ where $\text{Geom}(p)$ refers to a geometric distribution with parameter p.

We approximate the equivalent mass of a fragment with length L by taking $m_L = (L \times \overline{m}_{\text{unmodified}}) + \overline{m}_{\text{endtag}}$, where $\overline{m}_{\text{unmodified}}$ is the average mass of the unmodified nucleosides, and $\overline{m}_{\text{endtag}}$ is the average mass of the end labels.

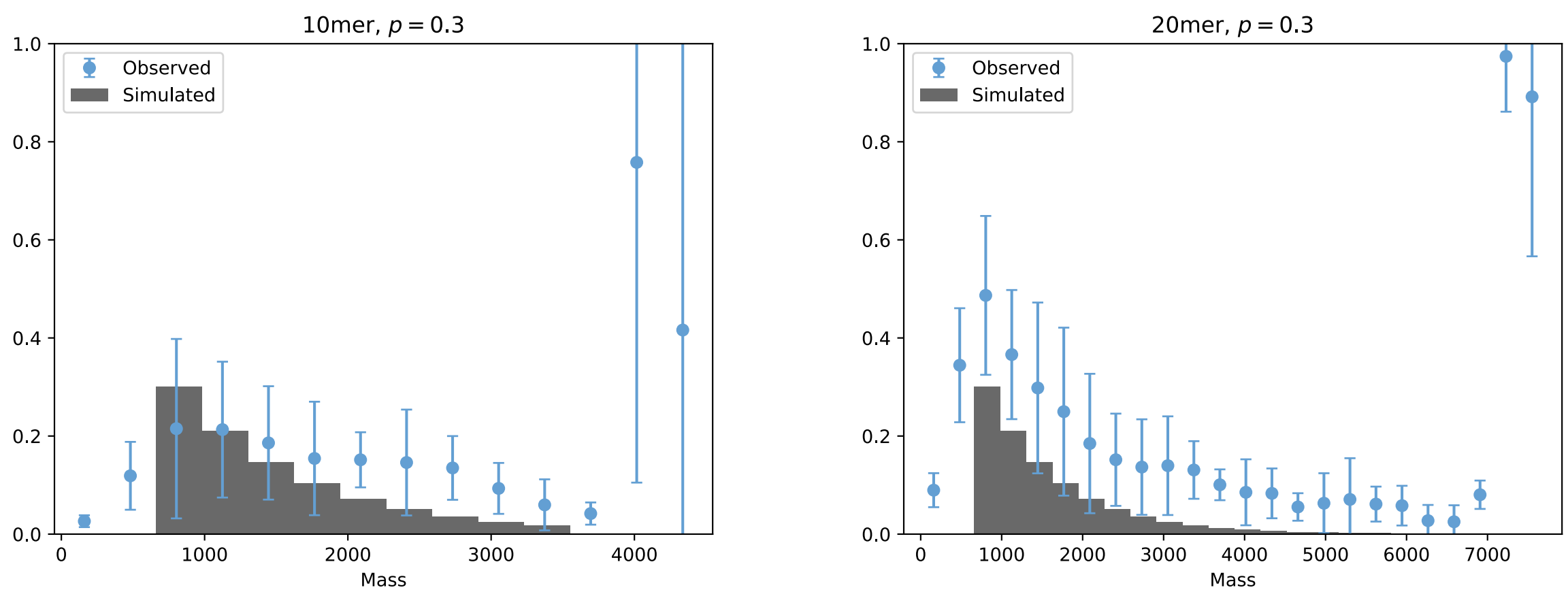

Figure S8: Comparisons of the observed and simulated fragment masses. Distribution of mean observed fragment mass, binned in intervals equivalent to $\overline{m}_{\text{unmodified}}$. Mean masses are taken over 5 samples of either a 10mer or 20mer RNA, with error bars equivalent to 1.96 times the standard deviation. The simulated fragment mass distribution is a geometric distribution with $p = 0.3$. The y-axis for the observed masses corresponds to a relative intensity, while for the simulated masses, it corresponds to the PMF of the geometric distribution.

Figure S8 compares the observed and simulated fragment mass distributions. The large peaks in the observed mass distribution correspond to the sequence mass. The simulated fragment masses taken from the geometric distribution with $p = 0.3$ qualitatively match the behavior of the distribution of the observed masses, hence the choice of this parameter in the simulation (Section Robustness of SpectrSeqTools on simulated data).

References

(1) Claesen, J.; Rockwood, A.; Gorshkov, M.; Valkenborg, D. *Mass Spectrometry Reviews* **2025**, *44*, 22–42.

(2) Liu, X.; Inbar, Y.; Dorrestein, P. C.; Wynne, C.; Edwards, N.; Souda, P.; Whitelegge, J. P.; Bafna, V.; Pevzner, P. A. *Molecular & Cellular Proteomics* **2010**, *9*, 2772–2782.

(3) Klein, J.; heckendorfc; Lukauskas, S.; armonyG; mstim Mobiusklein/Ms_deisotope: V0.0.53, Zenodo, 2024.

(4) Senko, M. W.; Beu, S. C.; McLaffertycor, F. W. *Journal of the American Society for Mass Spectrometry* **1995**, *6*, 229–233.

(5) Dittwald, P.; Valkenborg, D. *Journal of the American Society for Mass Spectrometry* **2014**, *25*, 588–594.

(6) Cappannini, A.; Ray, A.; Purta, E.; Mukherjee, S.; Boccaletto, P.; Moafinejad, S. N.; Lechner, A.; Barchet, C.; Klaholz, B. P.; Stefaniak, F.; Bujnicki, J. M. *Nucleic Acids Research* **2024**, *52*, D239–D244.
